# Gold Nanoparticle-Based Systems for Targeting the Ara h2 Allergen: A Molecular Modeling Approach

**DOI:** 10.34133/csbj.0088

**Published:** 2026-05-11

**Authors:** Aliyeh Mehranfar, Akhilesh Kumar Shakya, Roland Faller

**Affiliations:** ^1^ Department of Chemical Engineering, Texas Tech University, Lubbock, TX 79409, USA.; ^2^ Bioengineering, Whitacre College of Engineering, Texas Tech University, Lubbock, TX 79409, USA.

## Abstract

Arachis hypogaea 2 (Ara h2), the major peanut allergen responsible for severe allergic reactions in a large proportion of the population, is a key target for the detection of peanut allergenicity. In this study, the ability of gold nanoparticles functionalized with a peptide group to bind Ara h2 was investigated using molecular dynamics (MD) simulations. According to the structure of the peanut allergen Ara h2, which binds to a high-affinity IgE receptor, 130 amino acids of IgE have considerable interaction with the Ara h2 allergen. Therefore, a new peptide comprising these amino acids was designed as a functionalization agent for gold nanoparticles. To investigate the role of nanoparticle shape in the dynamics and interactions between Ara h2 and the designed systems, 2 shapes of gold nanoparticles were selected: rod-shaped (NR-Pep) and spherical (NP-Pep). Moreover, given the widespread use of phosphate-buffered saline (PBS) in experimental studies to mimic physiological conditions, its influence on the stability and behavior of the complexes was investigated alongside commonly used standard physiological conditions in MD simulations. MD simulations show that buffer conditions enhance the structural stability and compactness of the Ara h2@IgE complex. Based on these results, Ara h2 was substantially affected when interacting with functionalized gold nanoparticles (NP-Pep and NR-Pep). Among the studied nanoparticles, the rod (NR-Pep) forms a more stable complex with Ara h2 than do NP-Pep and IgE, which is the human receptor for the peanut allergen. Multiple analyses confirm that the designed nanoparticles are promising candidates for adsorbing the peanut allergen Ara h2.

## Graphical Abstract

**Figure:**
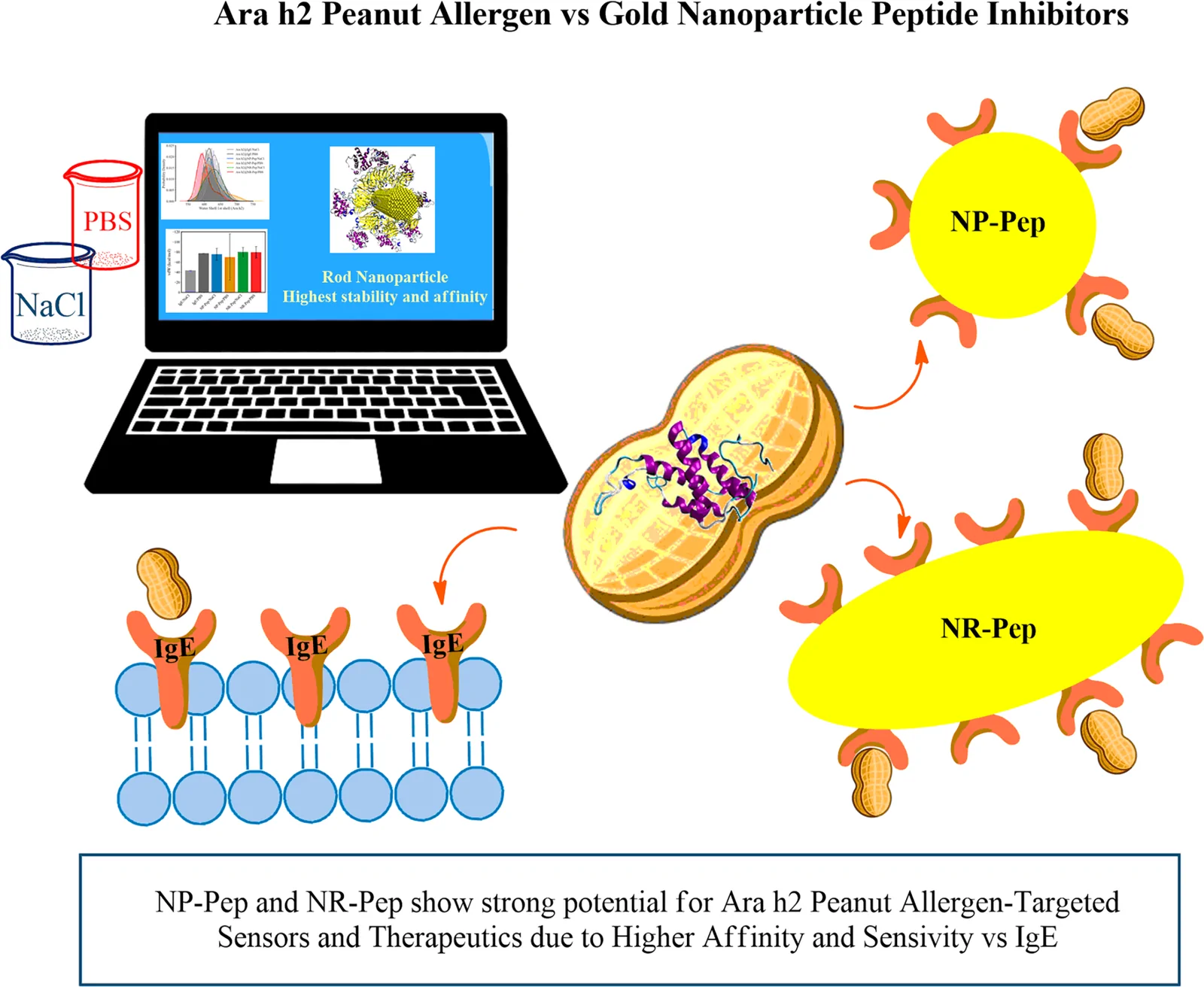

## Introduction

Food allergies, as one of the important health concerns rising over the past 3 decades, tend to be lifelong for about 80% of those affected. Among food allergies, peanut allergy is one of the leading causes of severe and sometimes fatal anaphylactic reactions [1–3]. Avoidance of allergens, along with the use of corticosteroids and antihistamines, are conventional approaches that provide only short-term relief of allergy symptoms but, in most cases, fail to address the root causes. In some cases, even 0.1 to 2 mg of peanut protein in foods can trigger symptoms [4,5]. Complete avoidance is extremely difficult, particularly since similar products frequently share production lines in food factories. Allergen-specific immunotherapy (AIT) has been explored as a treatment to induce tolerance against peanuts and provide long-term relief from allergic symptoms [6,7].

Recently, an oral allergen immunotherapy (OIT) (trade name PALFORZIA) was developed for rapid desensitization and received Food and Drug Administration (FDA) approval [8]. However, PALFORZIA can have adverse side effects [9]. In 2024, the FDA also approved another monoclonal antibody-based treatment, omalizumab, with some dietary restrictions to mitigate symptoms of peanut allergy in children [10,11]. Currently, using hypoallergenic proteins that eliminate immunoglobulin E (IgE)-binding domains, or selecting peptide regions capable of generating protective immune responses while avoiding IgE-binding epitopes, has become a successful trend in reducing the side effects of AIT [12,13]. Moreover, peptide antigens offer many advantages, including ease of production, scalability, and greater safety than whole proteins or crude protein extracts [14]. Since both surface accessibility and charge distribution significantly contribute to IgE molecular recognition of allergens [15], rationally designing peptide-based systems to block peanut allergen–IgE interactions can represent a promising therapeutic approach.

The World Health Organization (WHO) International Union of Immunological Societies (IUIS) Allergen Nomenclature Subcommittee has recognized 17 peanut-derived IgE-binding proteins as allergens, which vary clinically and immunologically across different regions of the world. Arachis hypogaea 2 (Ara h2) is recognized as the only major allergen consistently associated with peanut allergy across most populations [16,17]. The high stability of Ara h2, even at 100 °C, allows it to retain its allergenic potency, making it resistant to various food processing methods [18]. Ara h2 belongs to the 2S albumin family with a molecular weight of approximately 17 kDa, which contains cross-reacting conformational IgE epitopes. Therefore, Ara h2 is one of the potent targets for detecting peanut allergenic residues in the food industry [19]. The overall structure of Ara h2 encompasses 5 α-helical structures and a network of 4 conserved disulfide bridges [20]. Initial configurational explorations reveal 10 linear IgE epitopes on Ara h2, located in the N-terminal domain, the central region, and one in the C-terminal domain [21]. The allergenicity of proteins strongly depends on IgE epitopes. The linear and conformational epitopes were studied using different strategies, and epitope identification provides valuable information essential for understanding the antigen components involved in IgE cross-linking. Currently, predicting epitopes using in-silico methods provides fairly reliable results that are less time-consuming than exhaustive experimental approaches [22]. However, the contributions of Ara h2 epitopes to IgE-binding capacity remain unclear [23], and the potential relationship between structural features, particularly conformational epitopes, and the allergenicity of peanut proteins requires further substantiation.

Previous studies confirm the potential of nanotechnology for diagnosing and treating allergies, while also improving specificity and efficacy [24]. The unique physicochemical properties of nanomaterials make them applicable in a wide range of biomedical applications. They are easy to engineer for their size, shape, and morphology, which may significantly alter their performance [25–27]. Nanoparticles (NPs) can modulate the immune system to improve therapeutic efficacy and minimize side effects [28,29]. They can serve as carriers in nanomedicine, delivering therapeutic and diagnostic agents such as drugs and antibodies [30,31]. For instance, NP shapes play a significant role in cellular interactions [32]. Among different NP options, gold nanoparticles (AuNPs) have been applied successfully in nanobiotechnology and biomedicine. Their unique features make them advantageous for designing effective vaccines and generating high-affinity antibodies [33,34]. AuNPs have already been used as antigen carriers for subunit vaccines without producing anti-AuNP antibodies [35]. Few studies have confirmed the immunological responses of NPs, such as antibody and cytokine secretion [36,37]. One of the critical design parameters of NPs is their shape, which strongly influences how NP-based immunotherapies move through the bloodstream, are internalized by cells, and ultimately stimulate an immune response. Spherical AuNPs are among the most common candidates; the use of other forms, such as nanorods, has increased in recent years [38–42].

Investigation of immunological responses using different shapes of AuNPs for West Nile virus (WNV) antibody production has shown that spherical AuNPs generate the highest levels of WNV-specific antibodies, outperforming rod-shaped counterparts. Interestingly, rod-shaped AuNP-Es exhibit more efficient cellular uptake than spherical forms [42]. These findings highlight the impact of NP shape on immune activation, underscoring the importance of NP geometry in the design of effective nanomaterial-based vaccine platforms. Recently, peptide-functionalized AuNPs have been adopted in the pharmaceutical field as nanocarriers. Their large interacting surface, high specificity, and low toxicity make them advantageous [43,44]

Here, we present a novel approach using spherical and rod-shaped AuNPs conjugated with a peptide designed based on the IgE. The aim is to identify systems that bind Ara h2 and may modulate its interaction with IgE at the molecular level. In this work, we perform fully atomistic molecular dynamics (MD) simulations of the Ara h2 peanut allergen and investigate how Ara h2 interacts with the human IgE. This study reveals key IgE-binding sites on Ara h2, which help design peptide groups for AuNP functionalization. After identifying the candidate peptide, we apply a cysteine-capping strategy. The thiol (-SH) groups of cysteine anchor AuNPs through strong covalent gold–sulfur (Au–S) bonds, a common technique for functionalizing AuNP bioconjugates [45,46]. Once the AuNPs are functionalized with peptides, we examine the dynamic and interaction behavior of Ara h2 at these sites. By comparing the binding affinity of Ara h2 at the peptide-functionalized AuNP-binding site, we explore how receptor conformational changes influence these parameters. This approach provides insight for designing NP-based systems capable of binding peanut allergens.

## Methods

The Ara h2 structure [Protein Data Bank (PDB) ID: 8G4P] was obtained from the PDB database [47,48], with missing residues and disordered regions modeled using SWISS-MODEL [49,50]. The IgE structure (PDB ID: 2VXQ) [51,52] is a Fab fragment with 6 complementarity-determining regions (CDRs) that recognize antigens, as confirmed by the IMGT/3Dstructure-DB database [53,54]. For docking, Ara h2 was defined as the antigen, and the IgE Fab fragment as the antibody, to guide specific antibody–antigen interactions using ClusPro [55–57]. The Ara h2@IgE complex was then refined by MD simulations to achieve an equilibrated, structurally stable binding conformation. This equilibrated structure served as the basis for designing the peptide-functionalized NP systems.

AuNPs were designed using CHARMM-GUI [58], generating both a spherical gold nanoparticle (NP, diameter ≈ 4 nm) and a gold nanorod (NR, length ≈ 9 nm, diameter ≈ 4 nm). Surfaces were functionalized with a peptide derived from the antigen-binding region of the IgE using Discovery Studio [59]. After determining the most stable Ara h2@IgE conformation, the IgE structure was truncated to produce peptide segments, retaining all CDR regions and key connecting residues to preserve native 3-dimensional (3D) structure while ensuring suitability for NP coverage. The final selected sequence maintains the optimal construct while preserving the 3D organization of the binding interface needed for consistent interaction. To functionalize the AuNPs with a peptide group, cysteine residues were introduced at cleavage sites to enable peptide attachment to gold surface. This modification facilitates the formation of covalent sulfur–gold (Au–S) bonds, resulting in stable functionalization of the NP surface. The full sequence of the functionalized peptide (Pep) is as follows: VAL-GLN-LEU-LEU-GLU-SER-GLY-PRO-GLY-LEU-VAL-LYS-PRO-ALA-GLN-THR-LEU-SER-LEU-SER-CYS-ALA-VAL-SER-GLY-GLY-SER-ILE-ARG-SER-GLY-GLY-TYR-TYR-TRP-SER-TRP-ILE-ARG-GLN-HIE-PRO-GLY-LYS-GLY-LEU-GLU-TRP-ILE-GLY-TYR-ILE-TYR-HIE-SER-GLY-ASN-THR-TYR-TYR-ASN-PRO-SER-LEU-LYS-SER-ARG-ILE-ALA-MET-SER-VAL-ASP-THR-SER-GLU-ASN-LYS-PHE-SER-LEU-ARG-LEU-ASN-SER-VAL-THR-ALA-ALA-ASP-THR-ALA-VAL-TYR-TYR-CYS-ALA-ARG-LEU-ASP-GLY-TYR-THR-LEU-ASP-ILE-TRP-GLY-GLN-GLY-THR-LEU-VAL-THR-VAL-SER-CYS-GLU-ILE-GLU-MET-THR-GLN-SER-PRO-SER-SER-LEU-SER-ALA-SER-VAL-GLY-ASP-ARG-VAL-THR-ILE-SER-CYS-ARG-ALA-SER-GLN-ARG-ILE-ASN-THR-TYR-LEU-ASN-TRP-TYR-GLN-HIE-LYS-PRO-GLY-LYS-ALA-PRO-LYS-LEU-LEU-ILE-TYR-ALA-ALA-SER-SER-LEU-GLN-SER-GLY-VAL-PRO-SER-ARG-PHE-SER-GLY-SER-GLY-TYR-GLY-THR-ASP-PHE-THR-LEU-THR-ILE-SER-SER-LEU-GLN-PRO-GLU-ASP-PHE-ALA-SER-TYR-TYR-CYS-GLN-GLU-SER-LEU-SER-ALA-SER-TYR-THR-PHE-GLY-GLN-GLY-THR-LYS-VAL-GLU-ILE-LYS-ARG-THR-CYS

In total, 5 and 8 Pep groups were attached to the surfaces of the NP and NR, respectively, denoted as NP-Pep and NR-Pep, ensuring reasonable surface coverage while avoiding excessive steric crowding and prohibitive computational cost. Adding more than 5 peptides to the NP particle caused neighboring Pep groups to merge. For NR-Pep, adding more peptides significantly increased computational cost due to the large size of nanorod system. To maintain computational feasibility while preserving the 3D structure of the Pep, the number of peptides was limited to 8 for NR.

All MD simulations were performed using the Amber software [60,61]. The FF14SB force field [62] was used for proteins. Initial energy minimization was performed in 10,000 steps, followed by application of the *NVT* ensemble with the temperature raised to 310 K over 5,000 ps. Subsequently, *NPT* equilibration was performed for 10 ps, after which 500-ns *NPT* production MD simulations were performed with a 2-fs time step. Temperature and pressure were maintained constant at 310 K and 1 atm using the Langevin thermostat (2 ps^–1^ collision frequency) and the Berendsen barostat (1 ps relaxation time and compressibility of 4.5 × 10^–5^ bar^–1^), respectively [63–68]. All simulations were carried out under periodic boundary conditions (PBCs) in orthorhombic simulation boxes, and long range electrostatic interactions were treated using the particle mesh Ewald (PME) method with a 12-Å direct space cutoff [69]. The SHAKE constraint was applied to all bonds involving hydrogen atoms, and all the heavy atom bonds were treated without constraints [70]. Simulations were performed in water (TIP3P) [71,72] under physiological solution (150 mM NaCl), hereafter referred to as the NaCl solution. Also, the influence of phosphate-buffered saline (PBS) solvent is investigated, as it is widely used in experimental studies to mimic physiological conditions. The PBS buffer consisted of 0.137 M sodium chloride (NaCl), 0.0027 M potassium chloride (KCl), 0.01 M potassium dihydrogen phosphate (Na_2_HPO_4_), and 0.0018 M disodium hydrogen phosphate (KH_2_HPO_4_) [73,74]. Therefore, in addition to monovalent ions (K^+^, $H_{2}{\mathrm{PO}}_{4}^{-}$, Na^+^), the buffer environment contains divalent ${\mathrm{HPO}}_{4}^{2-}$ anions. Differences in ion composition, ionic strength, and the explicit presence of phosphate groups may significantly influence the system. In this environment, since KH_2_HPO_4_ and Na_2_HPO_4_ are not predefined as solvents, their molecules were explicitly added to achieve the target concentrations. AuNPs were modeled with force field parameters reported by Heinz et al. [75]. The S–Au bond parameters were taken from Groenhof and colleagues [76]. Studied systems are summarized in Table 1 with the number of binding sites, molecules, and residues for each simulation.

**Table 1.** Structural composition of each system with the number of binding sites, molecules, residues, and atoms

Systems	Solvent condition	No. of binding sites	No. of molecules	No. of residues	No. of atoms
Ara h2	NaCl solution	0	1	130	2,079
Ara h2@IgE	NaCl solution	1	3	556	8,477
Ara h2@IgE	PBS buffer	1	3	556	8,477
Ara h2@NP-Pep	NaCl solution	5	6	3,770	29,600
Ara h2@NP-Pep	PBS buffer	5	6	3,770	29,600
Ara h2@NR-Pep	NaCl solution	8	9	9,292	50,620
Ara h2@NR-Pep	PBS buffer	8	9	9,292	50,620

The molecular mechanics–Poisson–Boltzmann surface area (MM-PBSA) and molecular mechanics–generalized Born surface area (MM-GBSA) methods were utilized to estimate binding energy for the interactions between IgE and Ara h2 and the functionalized NPs and NRs from the final 100 ns of the production MD simulations [77,78]. Since entropic contributions were not explicitly included in the MM-GBSA analysis, the reported values represent approximate binding energy values. The extended simulation times ensured stable conformational sampling, and the stability of the systems was evaluated by monitoring structural properties such as root-mean-square deviation (RMSD) over the production trajectories, so independent replica simulations were not performed.

## Results and Discussion

### Dynamics of Ara h2

As one of the most potent allergens, Ara h2 represents an important target for addressing peanut allergy intervention. In this context, 500 ns MD simulations were employed to investigate the dynamical behavior and structural properties of Ara h2 under NaCl solution. Figure 1A shows the representative Ara h2 structure obtained after the initial simulation. To examine the structural stability of Ara h2 during simulation, several structural analyses were performed. The radius of gyration (Rg) is shown in Fig. 1B, with Rg values fluctuating between 15.9 and 16.6 Å . The average Rg value of 15.98±0.25 Å shows that the protein maintains its overall compactness and does not undergo major conformational changes. The stability of this protein was analyzed by the RMSD fluctuations (Fig. S1A). The obtained RMSD plot shows that the structure remains stable over the 500 ns simulation, with an average RMSD of 4.46±1.02 Å. The total solvent-accessible surface area (SASA) is also consistent with the Rg and RMSD analyses. The SASA plot (Fig. 1C) with fluctuations around ~8,500 to 9,500 Å^2^ confirms that the exposure of the protein surface to solvent remains relatively constant without affecting the hydrophobic cores. The same trend is observed from the polar SASA plot (Fig. S1C). In this analysis, the polar SASA values fluctuate around ~5,500 to 6,300 Å^2^, confirming the maintenance of the hydration shell. This behavior is commonly associated with stable protein–solvent interactions. Additional support for the structural stability of Ara h2 is provided by the end-to-end distance analysis (Fig. 1D).

**Fig. 1.**
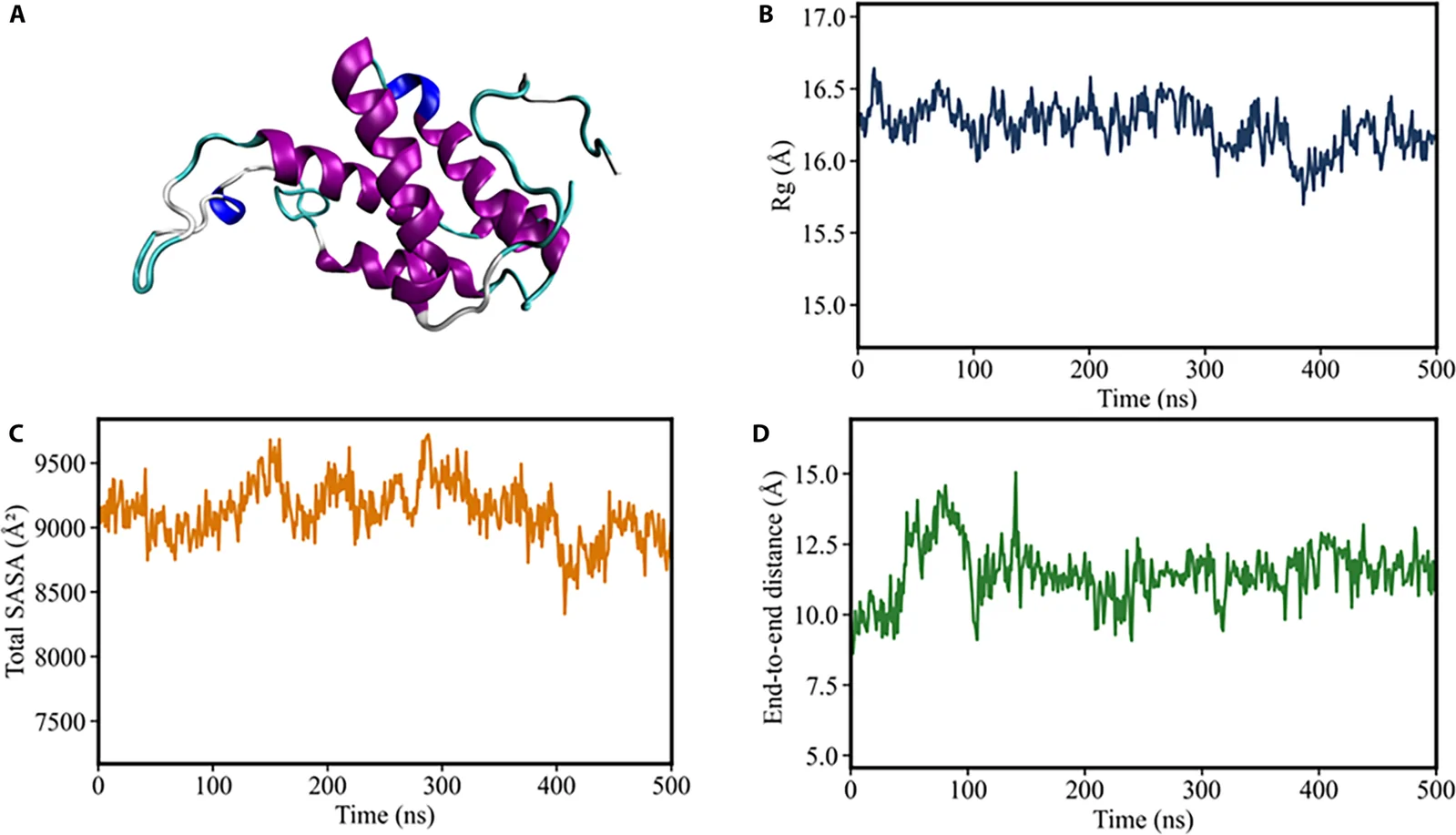
Structural dynamics results of Ara h2. (A) The 3D structure of Ara h2 after 500 ns of MD simulation. (B) Rg, (C) SASA, and (D) end-to-end distance plots of Ara h2 over the 500 ns MD simulation.

After an initial adjustment phase during the first ~100 ns, the end-to-end distance stabilizes around ~11 to 12 Å with moderate fluctuations, consistent with minor rearrangements of terminal and loop regions rather than large-scale structural changes. Local residue-level flexibility was further examined using root-mean-square fluctuation (RMSF) analysis (Fig. S1B). The RMSF profile reveals higher flexibility in residues approximately spanning positions 30 to 55 and near the C-terminal region. These residues correspond primarily to loop regions and terminal domains, which retain greater conformational freedom, whereas residues within the core region exhibit lower RMSF values, reflecting a more rigid and stable structural framework. The secondary structure analysis (Fig. S2) shows that the dominant α-helical content (green) remains well preserved throughout the trajectory, but the flexibility in the residues corresponding to the loop domains can be observed. These are the same residues that are identified as flexible in the RMSF analysis. The stability of the α-helical structure indicates that the protein maintains its native-like fold throughout the simulation. Overall, the long-term stability of Ara h2, along with localized flexibility around the loop and terminal domains, has been confirmed by 500 ns MD simulations under physiological conditions.

### Dynamics of Ara h2 at interaction with IgE in NaCl and PBS solutions

The interaction between Ara h2 and the IgE is directly linked to the clinical reactivity observed in peanut allergy [79,80]. Consequently, investigating the molecular interactions between Ara h2 and IgE can provide valuable insights for the rational design of novel nanostructure-based systems targeting this allergen. To this end, the dynamical behavior of Ara h2 in complex with IgE was examined under physiological and buffer conditions using the docked complexes obtained from the ClusPro docking platform. Figure 2 shows the obtained 3D structure of Ara h2@IgE at the end of simulation, in which several residues appear to have more contacts with specific IgE contact regions. A comprehensive structural analysis was performed to investigate the dynamics of Ara h2 interaction with IgE. The RMSD analysis (Fig. 2A) shows that, following an initial equilibration during the first ~200 ns, the Ara h2@IgE complexes reach stable conformations. The RMSD values increase in the PBS buffer (~2.5 to 3.0 Å), suggesting a more stable structure compared to NaCl solution. The Rg profiles (Fig. 2B) are in good agreement with the RMSD results. Although both systems maintain an overall folded architecture throughout the simulation, the buffer condition shows a more consistent Rg distribution, with reduced fluctuations compared to NaCl solution. This observation indicates a more conformationally restrained global structure in buffer, which may arise from electrostatic screening effects introduced by buffer ions that reduce long-range repulsive interactions at the Ara h2@IgE interface.

**Fig. 2.**
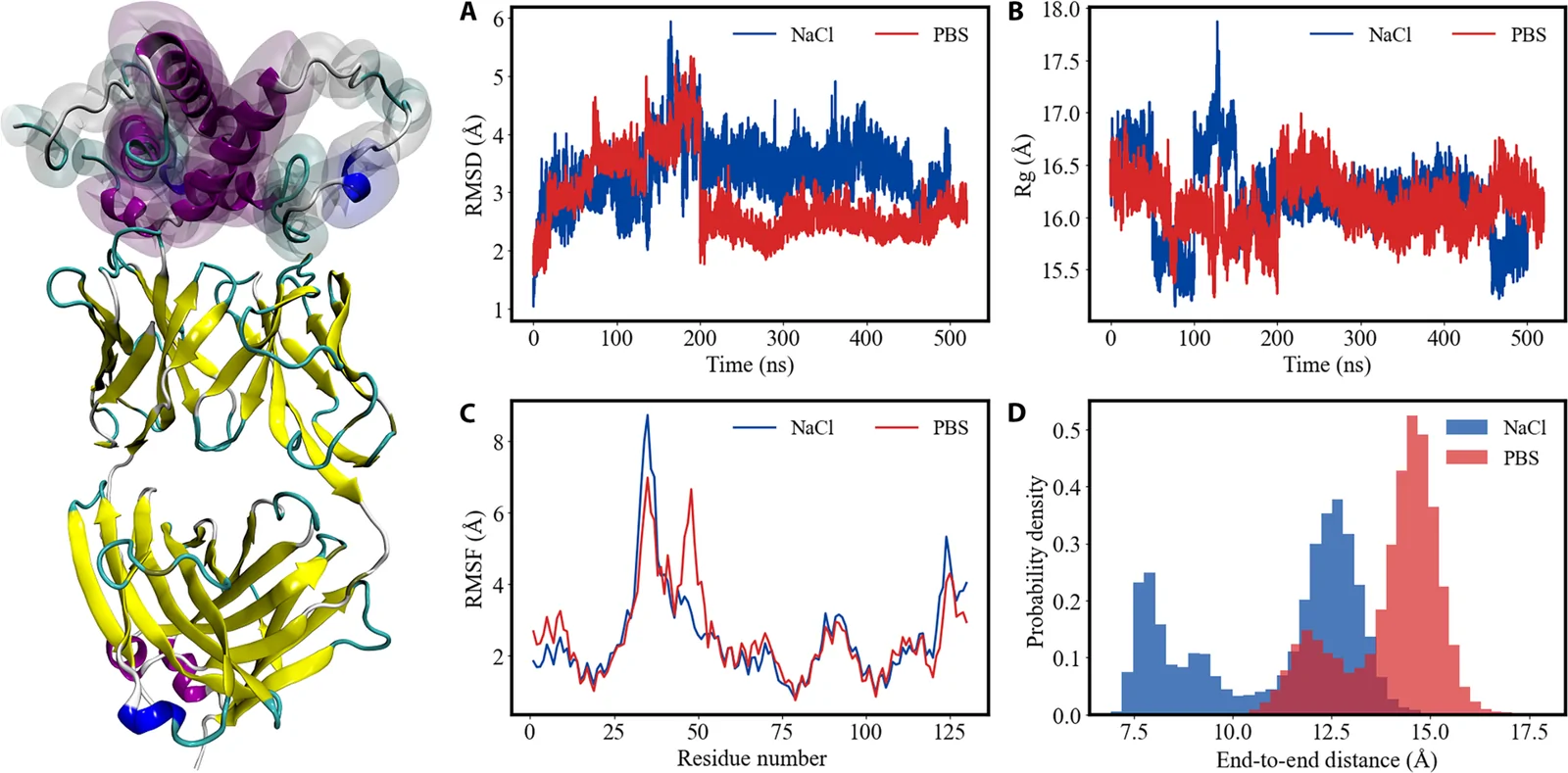
The 3D structure of the Ara h2@IgE and Ara h2 structural analysis in NaCl solution and PBS buffer conditions. (A) RMSD, (B) Rg, (C) RMSF, and (D) probability distribution of the end-to-end distance over the 500-ns simulations. NaCl solution and PBS buffer conditions are shown in blue and red, respectively.

The calculated RMSF profile (Fig. 2C) show that both systems exhibit elevated fluctuations in specific regions, consistent with the behavior observed for Ara h2 alone (Fig. S1B). These fluctuations are mainly associated with loop and terminal domains, which are known to participate in intermolecular recognition. Overall, Ara h2 residues display greater flexibility in NaCl solution than in PBS buffer. The highest RMSF values for Ara h2 in complex with IgE are observed at Tyr^35^, with maximum RMSF values of 8.71 Å in NaCl solution and 6.98 Å in buffer. Notably, Tyr^35^ also exhibits elevated flexibility in Ara h2 alone, with an RMSF value of 7.08 Å (Fig. S1B). The similarity between the isolated and buffer-bound states suggests that the buffer environment restricts local motions within functionally relevant regions. Such restrained flexibility may contribute to the formation of a more persistent and stable interaction interface between Ara h2 and IgE. The consistency in residue identity and flexibility across isolated and bound states highlights the intrinsically dynamic nature of this region and its potential role in mediating Ara h2@IgE interactions.

Ara h2 is a protein composed of 130 amino acids. In the Ara h2@IgE complex, residue numbering reflects the original numbering of the combined complex topology, in which Ara h2 residues correspond to positions 427 to 556. To ensure consistency and facilitate direct comparison with the isolated Ara h2 simulations, the residue numbering in the RMSF analysis was renumbered to reflect the native Ara h2 sequence (residues 1 to 130). In contrast, the secondary structure analyses retain the original residue numbering of the complex, as reported in this section for both Ara h2 and the IgE receptor.

The secondary structure analysis of Ara h2 in complex with IgE was performed for both studied conditions. As shown in Fig. 3, both environments preserve the dominant secondary structure features of Ara h2 throughout the simulations, indicating that complex formation does not induce large-scale unfolding. Under NaCl solution (Fig. 3A), Ara h2 maintains a high α-helical content across most of the simulation, particularly within the central regions of the protein. However, intermittent transitions to bends, turns, and coil-like conformations are observed, especially in loop and terminal domains.

**Fig. 3.**
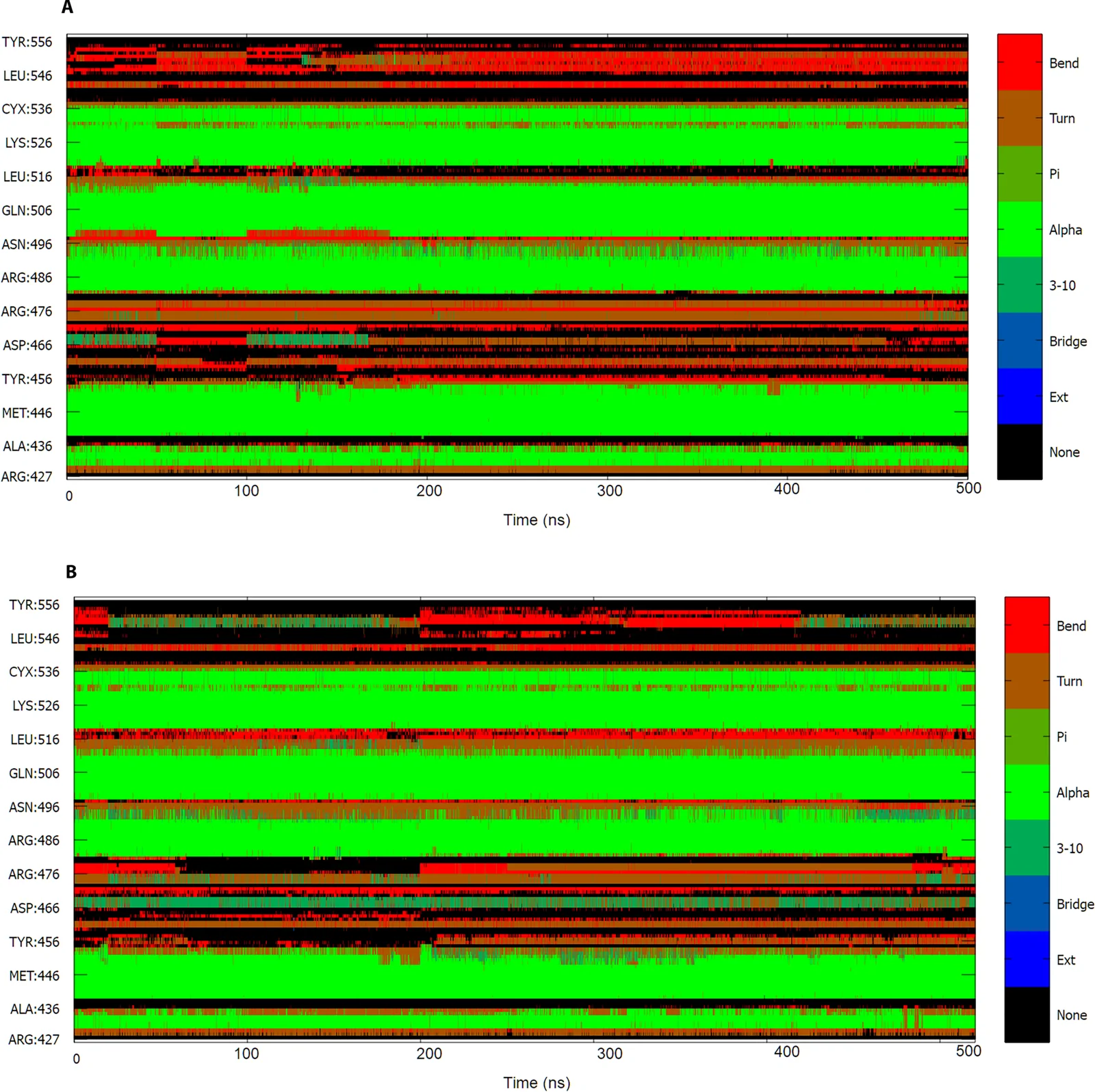
Secondary structure profiles of Ara h2 at the Ara h2@IgE complexes (A) under NaCl solution and (B) under PBS buffer conditions during 500 ns MD simulations. Colors indicate secondary structure types: α-helix (green), extended strand/β-sheet (blue), turn (brown), and bend (red).

The structural fluctuations are greater in NaCl solution, suggesting enhanced local flexibility and dynamic rearrangements in residues involved in intermolecular interactions. The more persistent patterns can be observed in the secondary structure profile (Fig. 3B) under buffer conditions. The α-helical domains remain notably stable, with fewer transitions into disordered states compared with NaCl solution. Regions that exhibit frequent structural switching under physiological conditions show reduced variability in buffer, indicating stabilization of local secondary structure elements. Collectively, these observations demonstrate that while the overall fold of Ara h2 is preserved in both environments, buffer conditions promote enhanced secondary structure stability and reduced structural plasticity, consistent with the RMSD and RMSF analyses.

The secondary structure analysis of the IgE was also examined under NaCl solution and buffer conditions (Fig. S3). In both environments, the IgE maintains a stable secondary structure, indicating that solvent conditions do not induce major structural rearrangements. Under NaCl solution (Fig. S3A), the receptor exhibits a well-preserved framework dominated by α-helical and extended conformations, with minor transitions into turns and bends, particularly in loop regions, which may facilitate intermolecular adaptability. In buffer conditions (Fig. S3B), secondary structure elements display increased temporal persistence, with fewer transitions between structural states. The α-helical regions remain stable throughout the simulation, while flexible regions show reduced variability. This behavior is consistent with electrostatic screening effects introduced by buffer ions, which dampen backbone fluctuations and stabilize hydrogen-bond networks. Consequently, buffer conditions promote a more dynamically restrained secondary structure behavior of the IgE, supporting enhanced stability of the Ara h2@IgE complex.

As a final structural analysis, the end-to-end distances of Ara h2 in the complexes with IgE were calculated and are shown in Fig. 2D. The average end-to-end distances of Ara h2 are 11.05±0.18 Å under NaCl solution, whereas a larger average value is observed in PBS (14.02±0.10 Å). In addition, analysis of the standard deviation (SD) shows that the end-to-end distance distribution in PBS is narrower (SD = 1.24 Å) than in NaCl (SD = 2.10 Å). Therefore, the Ara h2 protein in the Ara h2@IgE complex appears more extended and less conformationally heterogeneous in PBS buffer.

### Dynamics of Ara h2 while interacting with NP-pep and NR-pep

Structural analysis of Ara h2 and IgE shows 6 CDRs at the surface of the IgE receptor, which are able to interact with Ara h2; these can be considered the critical amino acids. Therefore, a new functional group based on IgE involving domains was designed and used to functionalize spherical and rod AuNPs. The length of the designed peptide is larger than that of conventional peptide inhibitors because the design strategy aimed to preserve the 3D arrangement of the IgE CDRs, rather than minimize sequence length. Truncation and shorter epitope-based designs resulted in loss of structural integrity and reduced binding stability. In this context, the peptide should be considered a structurally derived binding motif rather than a minimal linear peptide. Future work may focus on identifying shorter, optimized motifs that retain key interaction features.

Previous studies confirmed the performance of AuNPs for the detection of a specific IgE infood allergy testing [81,82]. To evaluate the performance of peptide-functionalized AuNPs as potential binding agents against the Ara h2 allergen, this section investigates the dynamical behavior of Ara h2 in complexes with the proposed functionalized AuNPs using MD simulations. The obtained complexes of the NP-Pep and NR-Pep with Ara h2 after 500 ns MD simulation are shown in Fig. 4A and B, respectively. These peptide-functionalized NPs present multiple binding sites on their surfaces, enabling multivalent interactions with multiple Ara h2 molecules. In contrast to the more localized binding observed in the IgE complex, Ara h2 can simultaneously bind multiple binding sites on the NP surface, leading to an avidity-driven interaction.

**Fig. 4.**
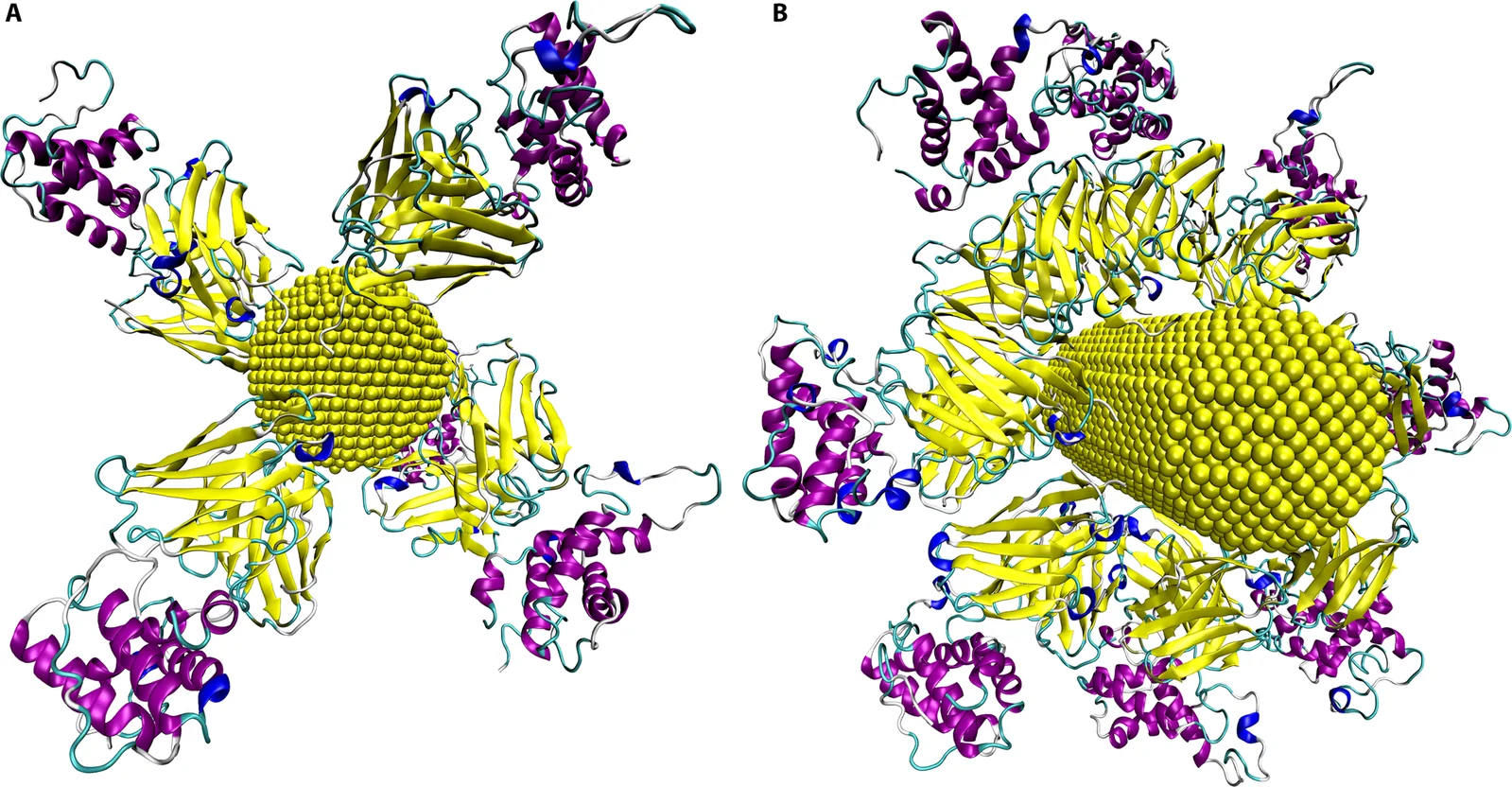
The 3D structures of the designed AuNP binding systems in NaCl solution. (A) Five Ara h2 are bound to individual binding sites in NP-Pep. (B) Eight Ara h2 are bound to individual binding sites in NR-Pep. Proteins are shown in cartoon representation (β-sheets in yellow, α-helices in purple, loops in cyan), and the nanoparticles are shown as a yellow sphere and rod.

All binding sites covered the interacting surface of Ara h2 and were therefore investigated in detail using structural analyses, motivating a systematic analysis of multiple binding configurations for each system. Trajectory analysis indicates that both NP-Pep and NR-Pep form stable complexes with Ara h2, exhibiting binding modes comparable to those observed for Ara h2 interaction with the human IgE. The relative orientations of Ara h2 with respect to NP-Pep and NR-Pep were compared across all binding sites, as shown in Figs. S4 to S7. In this analysis, the adsorbed Ara h2 structures were aligned using the Cα atoms of Ara h2 in the IgE-bound reference complex, enabling direct comparison of binding modes, with the reference structure highlighted in transparent grey. The results demonstrate that Ara h2 adopts a well-defined and consistent orientation in the IgE-bound complex (Figs. S4A and S6A). Multiple distinct orientations observed across both NP-Pep and NR-Pep systems indicate that Ara h2 interacts with the designed surfaces through diverse binding poses, reflecting greater configurational flexibility. In several configurations, Ara h2 maintains partial structural overlap with the IgE-bound conformation, whereas in other cases, significant deviations are observed. These findings suggest that peptide-functionalized AuNPs exhibit strong but structurally diverse binding modes, providing favorable binding environments without enforcing a single binding geometry comparable to that of the native IgE interaction. Therefore, these systems can act as high-affinity binding platforms rather than highly specific mimics of the IgE recognition interface.

To evaluate the stability and compactness of Ara h2 upon binding to the proposed systems, detailed RMSD and Rg analyses were performed for Ara h2 in complex with NP-Pep and NR-Pep under NaCl solution and buffer conditions. The full-time evolution of RMSD and Rg for all individual binding configurations is presented in Fig. S8. For the NP-Pep system, 5 distinct Ara h2-binding sites were examined, while for NR-Pep, 8 independent Ara h2 binding configurations were analyzed. All trajectories show that, after an initial equilibration phase, Ara h2 reaches a stable conformation and remains largely intact in the presence of both peptide-functionalized AuNP candidates over the 500 ns simulations. The Ara h2 conformational changes for each binding site are shown as lighter lines, with the average values indicated by solid lines.

The averaged RMSD and Rg profiles across all binding configurations are summarized in Fig. 5. The average RMSD (Fig. 5A) is lower in the Ara h2@NP-Pep complex (3.10±0.43 Å) than in NR-Pep (3.32±0.53 Å) under NaCl solution, which increases to 3.32±0.53 Å and 3.83±0.63 Å under buffer conditions, respectively. This increase indicates that the backbone structure adopts a slightly more relaxed arrangement in the buffer. The changes in Ara h2 compactness upon binding to the NPs are shown in the Rg plot (Fig. 5B). The average Rg value increases from 16.04±0.14 Å in NaCl solution to 16.59±0.15 Å in the buffer environment. Therefore, Ara h2 is more compact when binding to NP-Pep in NaCl solution than in buffer. On the other hand, for Ara h2@NR-Pep, the Rg values decrease from 16.03±0.18 Å in NaCl solution to 15.76±0.21 Å in buffer condition, suggesting more compactness in NaCl solution. The Rg observations are in agreement with the RMSD analysis, in which, after 350 ns MD simulations, the NP-Pep/ NaCl solution shows the lowest backbone deviation (3.10±0.43 Å). However, NP-Pep in the buffer, with an average RMSD of 4.11±0.48 Å, shows the highest RMSD, indicating greater conformational adaptation.

**Fig. 5.**
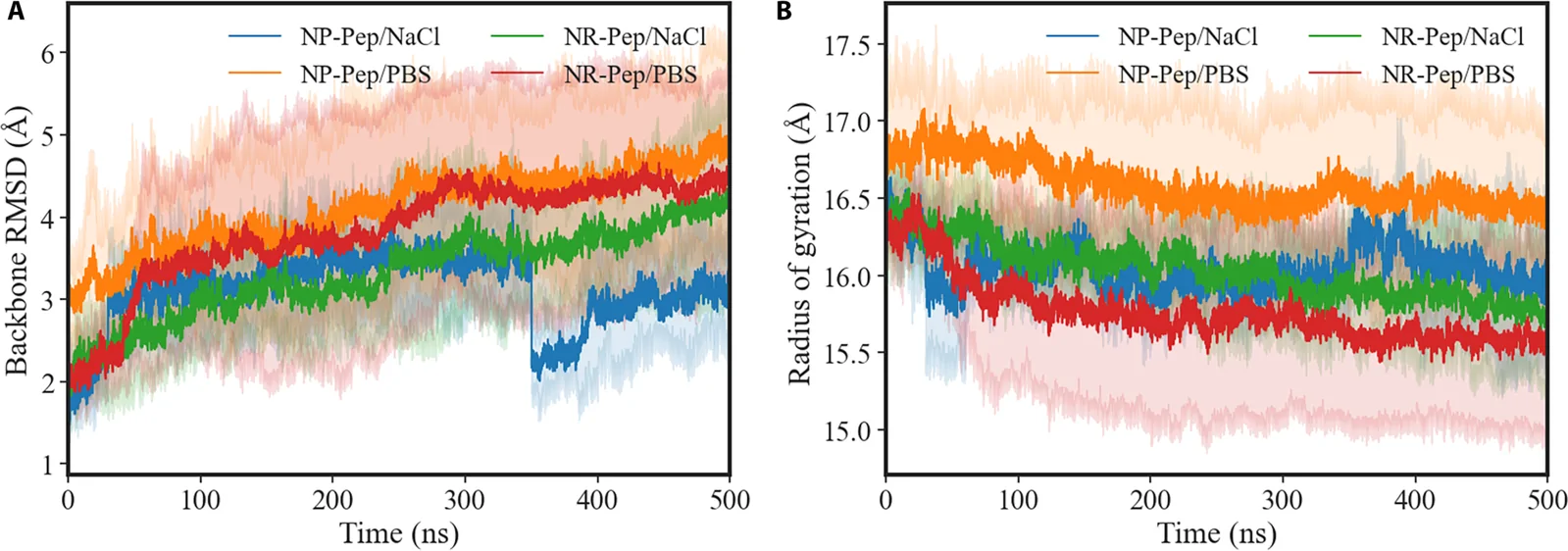
Structural analysis for the studied gold nanoparticle systems. (A) RMSD and (B) Rg profiles under NaCl solution and PBS buffer during 500 ns MD simulations. Solid lines highlight the average values across different Ara h2 binding situations.

Taken together, the detailed trajectory analysis (Fig. S8) and the averaged comparison (Fig. 5) demonstrate that both peptide candidates form stable complexes with Ara h2 across multiple binding configurations. However, clear differences emerge between NP-Pep and NR-Pep regarding their impact on Ara h2 dynamics. NP-Pep binding favors a more structurally stable Ara h2 conformation, particularly under NaCl solution, as reflected by lower backbone RMSD and relatively compact global dimensions. In contrast, NR-Pep binding induces greater conformational flexibility and backbone rearrangement, especially under buffer conditions, while simultaneously promoting partial global compaction of Ara h2. These results highlight the sensitivity of Ara h2 dynamics to both designed systems and solvent environment and provide important insights into how peptide-based designed NPs modulate Ara h2 structure in a manner comparable to its interaction with the human IgE.

The RMSF profiles correspond to 4 independent Ara h2 proteins interacting with different binding sites at NP-Pep and NR-Pep (Fig. S9). This allows us to assess the dynamics of this protein under NaCl solution and buffer conditions. All plots show that flexibility is not uniformly distributed along the sequence but instead localized to specific regions, indicating that Ara h2 maintains an overall stable fold while allowing localized motions. Profile A (Fig. S9A) shows the largest RMSF values around residues 260 to 264. Residues 260 to 264 in the full complex correspond to Ara h2 residues 31 to 35. The highest RMSF value is observed at residue 262 (8.15 Å) for Ara h2 in complex with NP-Pep under NaCl solution. Similar to Ara h2@NP-Pep, the maximum RMSF for Ara h2 in complex with NR-Pep is observed at residue 262 (7.12 Å) under NaCl solution, which reduces to 6.84 Å in buffer environment. Profile B (Fig. S9B) shows moderate but well-defined flexibility within the 589 to 718 region. For NP-Pep under NaCl solution, the highest RMSF value is observed at residue 618 (4.32 Å), corresponding to Ara h2 residue 30. However, under buffer conditions, the maximum peak shifts to residue 712 (4.95 Å), corresponding to Ara h2 residue 124.

For the NR-Pep systems, NaCl solution yields higher RMSF values at 619 (5.22 Å), 618 (5.07 Å), and 617 (4.41 Å) (Ara h2 residues 31 to 29). Buffer conditions further enhance localized flexibility at the C terminus, with peaks at 714 (5.75 Å), 712 (5.36 Å), and 713 (4.75 Å), corresponding to Ara h2 residues 124 to 126. These results indicate that this region displays solvent-sensitive flexibility, with buffer conditions redistributing motion toward the C-terminal domain of Ara h2. Profile C (Fig. S9C) is one of the examples of the strongest solvent-dependent behavior between all Ara h2 cases. Under NP-Pep/NaCl solution, exceptionally high RMSF values are observed at 975 (11.45 Å), 976 (11.25 Å), and 974 (9.68 Å) corresponding to Ara h2 residues 35 to 37. These values identify this domain as a major intrinsic flexibility hotspot. In buffer, these fluctuations are substantially suppressed, with the highest RMSF values reduced to 1,071 (5.38 Å), 982 (5.19 Å), and 1,070 (4.58 Å), mapping to Ara h2 residues 130, 41, and 129, respectively. For the NR-Pep systems, NaCl solution shows moderate flexibility at 976 (4.91 Å), 975 (4.86 Å), and 974 (4.40 Å) (Ara h2 residues 36 to 34). Under buffer conditions, partial restoration of flexibility is observed at 975 (6.27 Å), 1,065 (5.82 nm), and 976 (5.81 Å), corresponding to Ara h2 residues 35, 124, and 36. Overall, this region emerges as a highly dynamic domain of Ara h2 whose flexibility is strongly modulated by the solvent environment. In profile D (Fig. S9D), localized flexibility is primarily observed around residues 1,331 to 1,333. For NP-Pep under NaCl solution, the largest RMSF values occur at 1,332 (5.34 Å), 1,333 (5.27 Å), and 1,331 (4.67 Å), corresponding to Ara h2 residues 34 to 36. Buffer conditions induce a pronounced increase in flexibility, with dominant peaks at 1,341 (8.07 Å), 1,340 (6.58 Å), and 1,337 (4.65 Å), mapping to Ara h2 residues 43 to 39. In contrast, NR-Pep systems display more restrained fluctuations in this region. Under NaCl solution, RMSF peaks are observed at 1,422 (4.66 Å), 1,332 (4.48 Å), and 1,333 (4.38 Å), corresponding to Ara h2 residues 124 and 34 to 35. Buffer conditions show similar but slightly reduced fluctuations at 1,332 (4.89 Å), 1,333 (4.79 Å), and 1,331 (4.31 Å), again mapping to Ara h2 residues 34 to 36.

Overall, the RMSF analysis shows that Ara h2 exhibits region-specific intrinsic flexibility, with the most prominent fluctuations localized to residues 30 to 36 and 124 to 130 across all 4 Ara h2 proteins. NaCl solution generally promotes higher mobility of residues, whereas buffer conditions dampen fluctuations and, in some regions, redistribute flexibility along the sequence. These findings establish a quantitative baseline for Ara h2 intrinsic dynamics and provide a critical reference for interpreting changes in flexibility observed upon peptide or IgE binding.

### Ara h2 interactions with IgE, NP-pep, and NR-pep

Affecting the dynamics of Ara h2 while interacting with different binding sites may cause a change in the type and nature of interactions of this protein with receptor-binding sites. As shown in Fig. 6A, the SASA values have the largest total when Ara h2 is bound to IgE, suggesting a more solvent-exposed configuration. The total SASA in NaCl solution has an average of 8,058±331 Å^2^, while it shifts to lower values in PBS buffer (7,734±531 Å^2^), indicating reduced solvent exposure under buffer conditions. Binding to bothNP-Pep and NR-Pep reveals a reduction in total SASA relative to IgE. The average values of total SASA while interacting with NP-Pep reduce to 7,642±385 Å^2^ and 6,791±787 Å^2^ in NaCl solution and PBS buffer, respectively. In the case of NR-Pep, the average total SASA is observed at 7,732±385 Å^2^ in NaCl solution, which decreases modestly to 7,555±457 Å^2^ in buffer. In total, NP-Pep exhibits the lowest overall SASA, particularly under buffer conditions, indicating extensive surface masking and increased conformational heterogeneity.

**Fig. 6.**
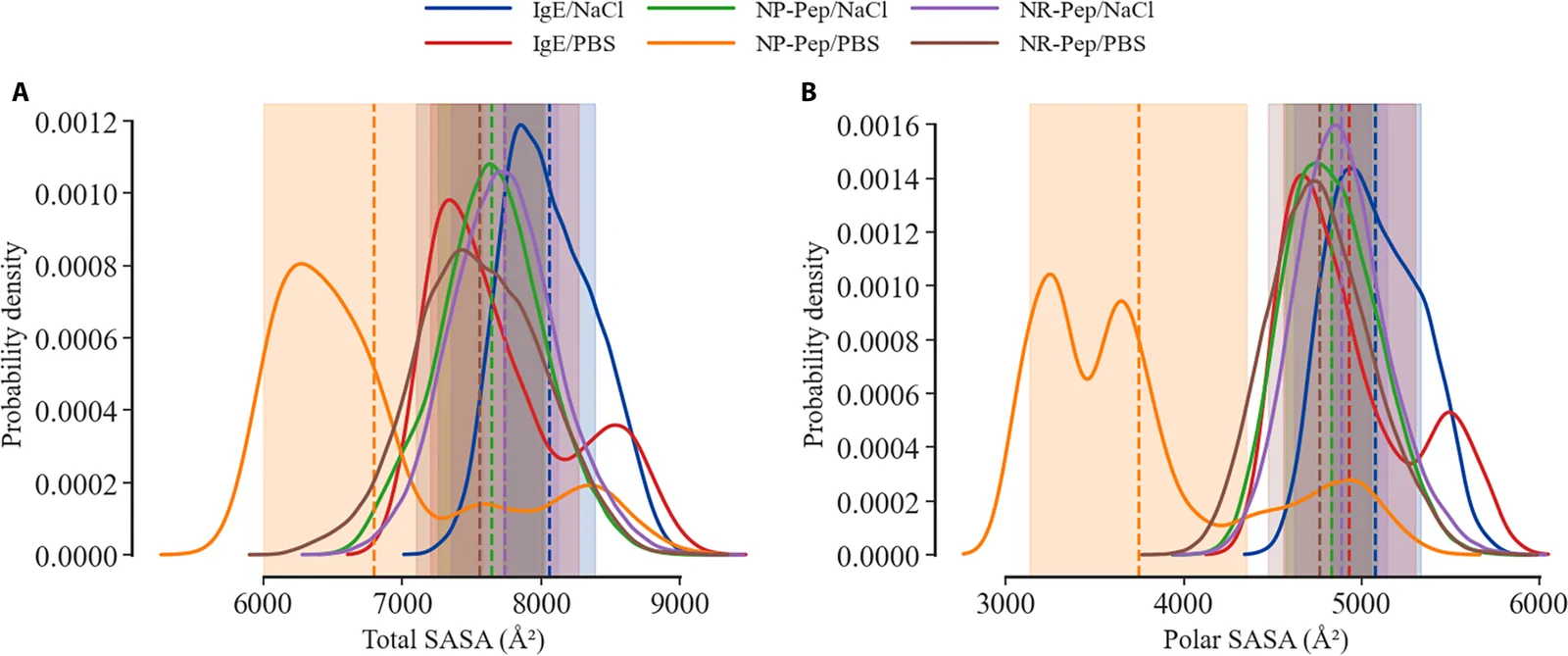
Total and polar SASA distributions of Ara h2. Probability density distribution profiles of (A) total SASA and (B) polar SASA of Ara h2 in complex with IgE, NP-Pep, and NR-Pep under NaCl solution and PBS buffer conditions. Dashed vertical lines indicate the average SASA values for each system, while shaded regions highlight the corresponding distribution ranges.

The largest reduction in SASA is observed when Ara h2 binds to NP-Pep-binding sites. These conclusions complement the RMSD, Rg, and RMSF analyses, highlighting that binding approaches by NP-Pep and NR-Pep modulate the surface properties of Ara h2 relative to its interaction with the IgE.

To characterize binding patterns and assess how solvent conditions influence the solvation environment of Ara h2, the distributions of water molecules in the first and second hydration shells were analyzed for Ara h2 bound to IgE, NP-Pep, and NR-Pep under NaCl solution and buffer conditions. Figure 7 presents the probability distributions of hydration shell water populations around Ara h2 for the different systems. The hydration shell analysis reveals that Ara h2 exhibits the highest local and extended hydration when bound to IgE, particularly under NaCl solution. The average number of water molecules in the first (636.25±21.06) and second (1,046.68±30.60) hydration shells in a NaCl solution decreases to 615.57±17.33 and 1,004.54±26.49, respectively, under a PBS buffer environment. This reduction confirms that local hydration at the Ara h2 surface is reduced in the buffered environment, which is consistent with a more compact or reorganized solvent-accessible surface under buffer conditions. For improved clarity, the first-shell hydration distributions of Ara h2 when bound to IgE are additionally presented in Fig. S11A.

**Fig. 7.**
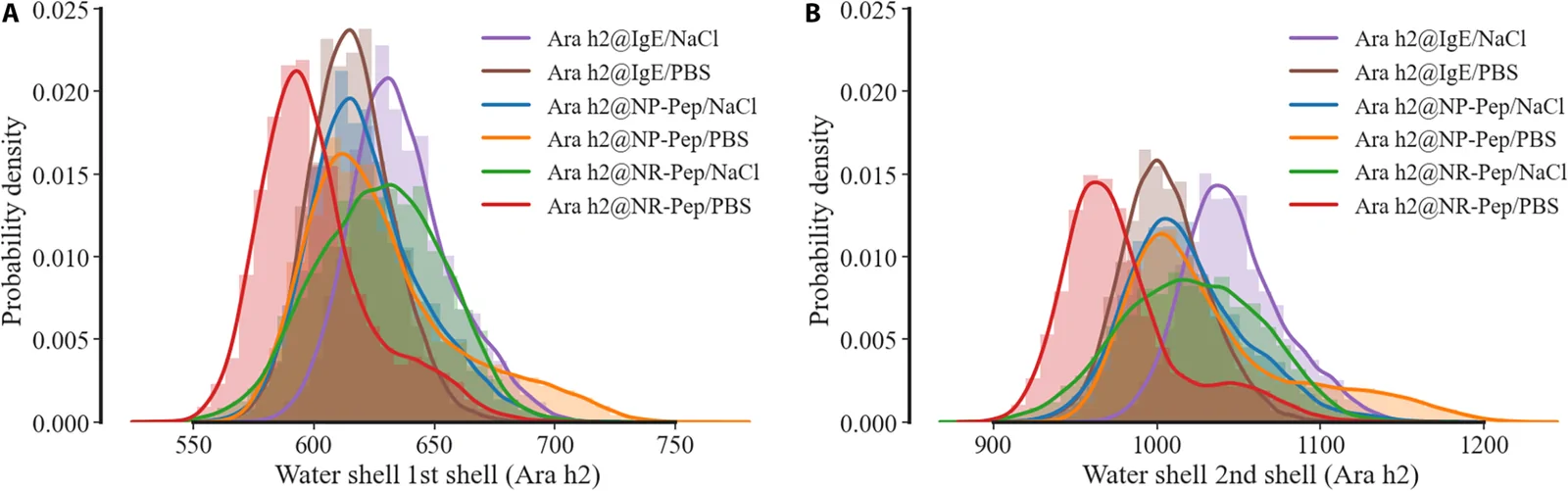
Water shell density distributions around Ara h2. Probability density distributions of the number of water molecules: (A) first hydration shell and (B) second hydration shell around Ara h2 in complex with IgE, NP-Pep, and NR-Pep under NaCl solution and PBS buffer conditions.

Binding of Ara h2 to the designed peptide-functionalized NPs further alters both hydration shells, with buffer conditions generally reducing the number of associated water molecules. However, the average number of water molecules around Ara h2 decreases in the buffer when it is bound to both NP-Pep and NR-Pep. The most significant reduction in hydration is observed for Ara h2@NR-Pep under buffer conditions. The Ara h2@NP-Pep maintains intermediate hydration levels, with average values of 628.15±31.88 (first shell) and 1,033.01±52.41 (second shell) under buffer conditions. The observed reduction in first- and second-shell hydration upon binding to the designed systems, particularly under buffer conditions, is consistent with the decreased total and polar SASA, reflecting reduced solvent accessibility of Ara h2 surface regions due to peptide-induced surface masking and surface reorganization. These hydration trends are further supported by RMSD, Rg, and RMSF analyses, which collectively demonstrate that NP-Pep and NR-Pep modulate the surface properties and solvent interactions of Ara h2 in a manner distinct from its native interaction with the IgE.

The calculated averages of interfacial contacts are shown in Fig. S10. The minimum number of contacts is observed when Ara h2 is bound to IgE in NaCl solution (907.99±187.96), which increases to 1,460.66±220.45 under buffer conditions. This increase is consistent with the reduced SASA and hydration shell populations observed in buffer, indicating partial surface reorganization and reduced solvent accessibility. In contrast, Ara h2 forms a substantially larger number of contacts when bound to the designed peptide-functionalized NPs, suggesting stronger interfacial engagement compared with IgE.

The maximum number of contacts is observed for Ara h2 bound to NP-Pep in buffer, with an average of 2,040.87±1,451.71, suggesting extensive interfacial reorganization and surface masking of Ara h2. NR-Pep shows an average number of contacts of 1,775.67±478.41 in NaCl, decreasing slightly to 1,513.10±575.59 in buffer, indicating a more restrained interaction compared with NP-Pep in the same environment. Overall, these contact number trends support the conclusion that NP-Pep induces the most extensive surface masking and interfacial reorganization of Ara h2, particularly under buffer conditions, consistent with buffer-mediated electrostatic screening and enhanced nonpolar packing. Together with the SASA, hydration shell, RMSD, Rg, and RMSF analyses, these results highlight distinct modes by which the designed systems modulate Ara h2 surface properties relative to its native interaction with the IgE.

Hydrogen-bond (H-bond) interactions play an important role in protein stability and in mediating interactions at binding interfaces; therefore, hydrogen-bonding analysis was performed. The hydrogen-bonding distributions for Ara h2 bound to the IgE under NaCl solution and buffer conditions are summarized in Fig. S11B and Table S3. The average number of H-bonds formed by Ara h2 increases from 1.68±1.26 under NaCl solution to 3.40±2.41 in buffer. Unlike NaCl solution, the distribution is broader and extends to higher hydrogen-bond numbers under buffer conditions. This enhancement is consistent with increased ionic strength and altered solvent organization around the protein surface, which can promote additional transient hydrogen-bonding interactions. To enable comparison of hydrogen-bond interactions not only with IgE but also with the designed systems, the average number of hydrogen bonds is reported in Fig. 8.

**Fig. 8.**
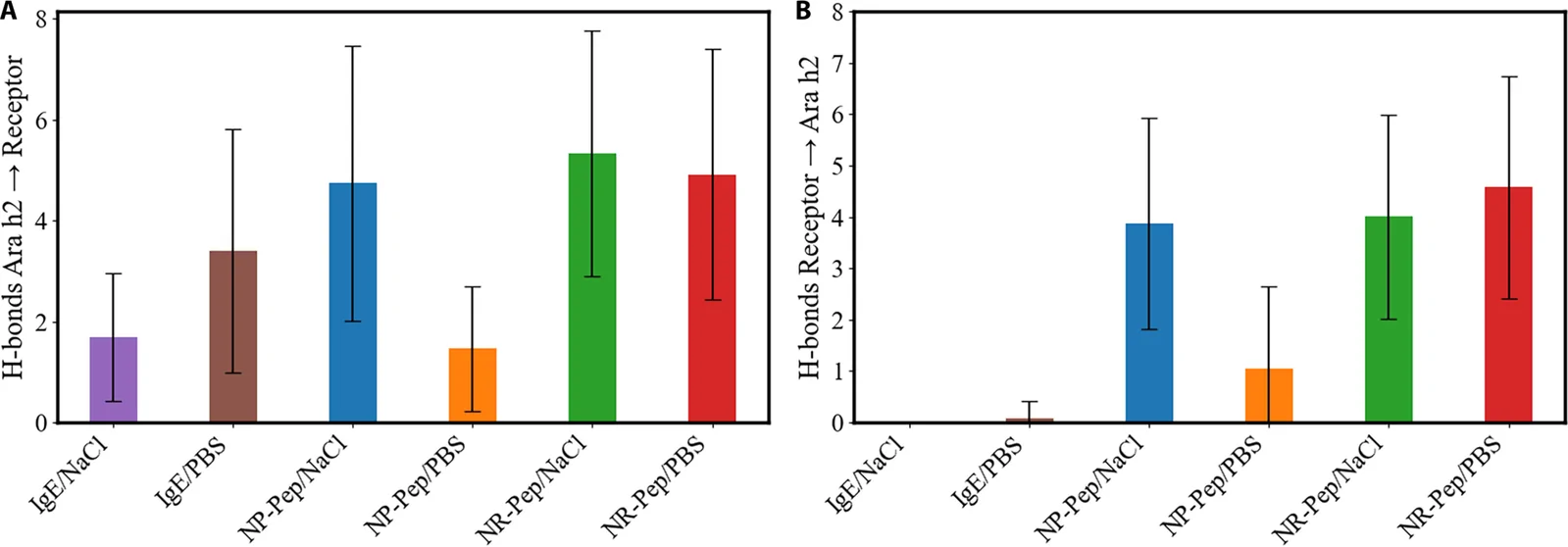
Average numbers of H-bonds between Ara h2 and its binding receptors. (A) Average number of H-bonds donated by Ara h2 to the receptor (Ara h2 → receptor). (B) Average number of H-bonds donated by the receptor to Ara h2 (receptor → Ara h2) under NaCl solution and PBS buffer conditions over the 500 ns MD simulations.

The analysis shows H-bonds donated by Ara h2 to the binding site (Ara h2 → receptor; Fig. 8A) and those donated by the receptor to Ara h2 (receptor → Ara h2; Fig. 8B). For the Ara h2@IgE complex, the average number of H-bonds in the receptor → Ara h2 direction is relatively low and negligible under both NaCl solution and buffer conditions, indicating that H-bonds play a limited role in stabilizing the Ara h2@IgE interaction, particularly under NaCl solution. The average number of hydrogen bonds increases markedly when Ara h2 interacts with the designed systems compared with IgE. Under NaCl solution, Ara h2 donates an average of 4.74±2.72 hydrogen bonds to NP-Pep while receiving 3.88±2.06 hydrogen bonds from this NP system. Both values decrease substantially under buffer conditions, to 1.46±1.23 (Ara h2 → NP-Pep) and 1.04±1.60 (NP-Pep → Ara h2). These results indicate that NP-Pep binding is strongly hydrogen-bond-driven under physiological conditions, whereas buffer conditions reduce the contribution of direct hydrogen bonding, likely due to increased ionic screening and partial replacement of hydrogen bonds by electrostatic interactions (ElEs) and van der Waals (vdW) interactions.

Despite the strong influence of NP-Pep and IgE on the solvent environment, NR-Pep reveals a strong, persistent hydrogen-bonding behavior across solvent environments. In summary, NR-Pep accepts 5.32±2.43 and donates 4.01±1.98 H-bonds from and to Ara h2 proteins. These values decrease slightly in buffer, to accepting 4.91±2.48 and donating 4.57±2.16. This relative insensitivity to solvent conditions indicates that NR-Pep forms a balanced H-bond network with Ara h2 that is less sensitive to ionic conditions. Receptors such as IgE and the designed peptide-functionalized NPs can associate with Ara h2 through a combination of ElE and vdW interactions. Therefore, the ElE and vdW interaction energies between Ara h2 and the receptors were quantified using the linear interaction energy (LIE) analysis method [83]. The frequency distributions of ElE and vdW interactions for the Ara h2@IgE complex under NaCl solution and buffer conditions are shown in Fig. S11, with the corresponding statistical values summarized in Table S3. Both the ElE energy (Fig. S11C) and the vdW energy (Fig. S11D) profiles exhibit a clear stabilization effect under buffer conditions.

The LIE analysis shows that the average ElE energy for the Ara h2@IgE complex changes from −241.52±56.96 kcal/mol in NaCl solution to a more favorable −284.11±57.66 kcal/mol in buffer. The vdW energy profile reveals a similar trend, although with a smaller magnitude. The average vdW energy shifts from −43.50±9.32 kcal/mol in NaCl solution to −77.41±8.04 kcal/mol in buffer. Regarding the greater contribution of ElE energy relative to vdW, ElE plays a more important role in stabilizing Ara h2@IgE complexes. The observed enhancement in ElE interactions in the buffered environment is consistent with increased ionic strength and screening of long-range charge–charge interactions. In addition, the narrower vdW energy distribution observed under buffer conditions implies more persistent, optimized nonpolar packing at the interface.

The investigation of ElE and vdW interaction energies was extended to elucidate the energetic driving forces governing Ara h2 recognition by the designed peptide-functionalized AuNPs. Figure 9 presents the system-averaged interaction energies, obtained by averaging contributions across all Ara h2s for each complex. In contrast, the interaction energies resolved for individual binding sites are summarized in Figs. S12 and S13. In these analyses, IgE exhibits a single binding interface for Ara h2 adsorption, whereas NP-Pep binds Ara h2 structures through 5 distinct binding sites, and NR-Pep binds through 8 distinct binding sites, each evaluated separately. This analysis enables direct comparison between site-specific interaction patterns and the global energetic behavior of each Ara h2@receptor complex. The corresponding ElE and vdW values for each specific complex are summarized in Table S4.

**Fig. 9.**
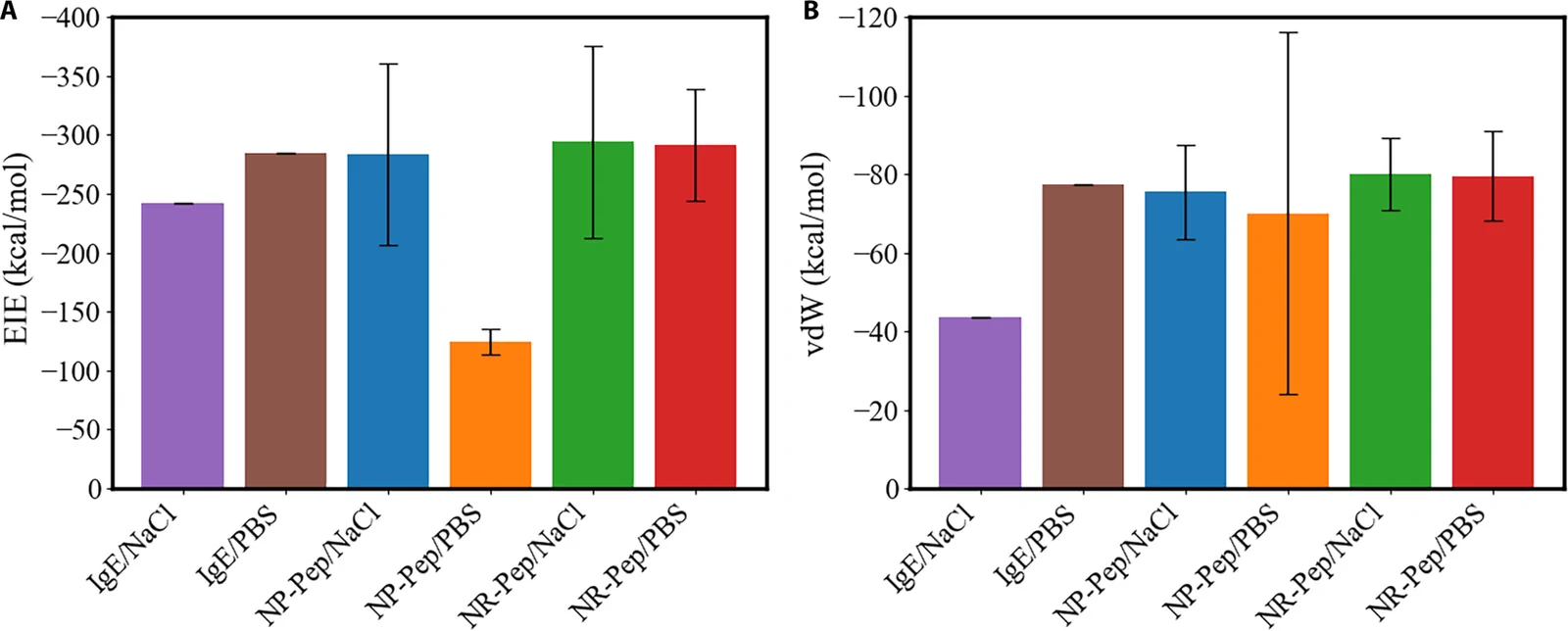
Ara h2@receptor interaction energies. The average electrostatic (ElE) and van der Waals (vdW) interaction energies between Ara h2 and its binding receptors (IgE, NP-Pep, and NR-Pep) under NaCl solution and PBS buffer conditions, calculated using the linear interaction energy (LIE) approach. Error bars denote standard deviations.

Among all candidates, NP-Pep exhibits the strongest sensitivity to solvent conditions. The site-resolved ElE profiles (Fig. S12) show that NP-Pep under buffer conditions displays the weakest ElE stabilization compared with the other designed systems. The system-averaged ElE interaction energy decreases markedly from −283.38±76.65 kcal/mol in NaCl solution to −124.85±10.77 kcal/mol in buffer, highlighting a pronounced solvent-dependent weakening of ElE interactions. At the site level (Fig. S12 and Table S4), this solvent sensitivity is also evident. The most favorable Ara h2@NP-Pep complex exhibits an ElE energy of −379.14±95.44 kcal/mol under physiological conditions. The least one forms under buffer conditions, with an ElE of −110.05±67.04 kcal/mol. Therefore, the buffer environment can disrupt the ElE interactions between NP-Pep and Ara h2. The vdW contributions for NP-Pep follow trends similar to those of ElE, although their overall magnitude is lower. The system-averaged vdW interaction energy (Fig. 9B) is −75.44±12.09 kcal/mol under NaCl solution and becomes slightly less favorable in buffer (−70.02±46.13 kcal/mol), indicating a modest solvent dependence of nonpolar interactions. At the site level (Fig. S13 and Table S4), the most favorable vdW interaction under buffer conditions is observed at −109.78±0.50 kcal/mol, which weakens substantially in NaCl solution to −72.69±9.42 kcal/mol. Conversely, the weakest vdW interaction in buffer occurs at Ara h2_5 (−6.15±0.38 kcal/mol), which becomes significantly more favorable in NaCl solution (−69.09±8.06 kcal/mol). In NaCl solution, the strongest vdW stabilization is observed at Ara h2_2 (−96.18±20.34 kcal/mol).

Consistent with other observations for NR-Pep, the ElE and vdW interaction energies exhibit overall stability across the studied solvent environments. The system-averaged ElE values (Fig. 9A) remain very similar in NaCl solution (−293.88±81.61 kcal/mol) and buffer (−291.80±47.34 kcal/mol), indicating a largely solvent-insensitive ElE stabilization mechanism at the global level. However, inspection of the site-resolved ElE profiles in Fig. S12 reveals notable variability among individual Ara h2@NR-Pep-binding sites. For example, at the first binding site, the ElE becomes more favorable upon transitioning from NaCl solution (−217.56±52.47 kcal/mol) to buffer (−371.85±100.60 kcal/mol).

In contrast, the second binding site exhibits the opposite trend, with ElE changing from −388.01±66.92 kcal/mol in NaCl solution to −251.69±72.23 kcal/mol in buffer. One of the Ara h2 complexes with NR-Pep, with an ElE energy of −436.16±72.94 kcal/mol in NaCl solution, is the most favorable complex compared to all the studied complexes. However, this complex becomes less favorable in buffer (−356.05± 78.43 kcal/mol). The weakest ElE interaction occurs at Ara h2_3 (−185.39±49.93 kcal/mol) in NaCl solution, while becoming stronger under buffer conditions (−251.40±43.23 kcal/mol).

Despite pronounced site-specific differences, the opposing solvent responses largely compensate when averaged across all binding sites, yielding comparable system-level ElE stabilization in both environments. This behavior highlights that NR-Pep binding is characterized by heterogeneous local ElE interactions but robust global energetics. A very similar trend is observed for the vdW contributions (Fig. 9B). The average vdW energies of Ara h2@NR-Pep complexes are very similar under physiological (−80.09±9.18 kcal/mol) and buffer (−79.51±11.41 kcal/mol) conditions, reflecting persistent nonpolar packing at the interaction interface. At the site level, the strongest and weakest vdW interactions have values of −103.69±16.48 kcal/mol and −67.39±11.19 kcal/mol, respectively, both under buffer conditions. However, strong vdW interactions are also observed in NaCl solution, such as in a complex with a vdW energy of −101.49±19.05 kcal/mol.

Overall, based on the calculated ElE and vdW interaction values, ElE interactions are more important for the stability of Ara h2 complexes with different receptors. Moreover, it can be concluded that Ara h2 bound to NR-Pep under NaCl solution exhibits the most favorable binding, whereas Ara h2 bound to NP-Pep under buffer conditions shows the weakest binding. These results reveal 2 distinct binding strategies employed by the designed systems.

The energy analysis confirms that interactions between Ara h2 and its receptors induce changes in its dynamical behavior and structural stability. Based on this, NR-Pep engages Ara h2 through a balanced combination of ElE, vdW, and hydrogen-bond interactions, preserving moderate solvent accessibility, stable hydration, and consistent energetic stabilization across conditions. But the NP-Pep system promotes extensive surface masking and interfacial reorganization of Ara h2, achieving high contact density and strong solvent-dependent modulation of ElE interactions, but at the cost of increased heterogeneity and sensitivity to the solvent environment. Therefore, based on the energy analysis, the interaction between Ara h2 and receptors alters the dynamical behavior and structural stability of Ara h2.

### Binding energy

The relative interaction strengths of Ara h2 with the IgE, NP-Pep, and NR-Pep systems were evaluated using MM-GBSA analysis. Because entropic contributions were not explicitly calculated, the reported values should be regarded as estimates of binding energy. The MM-GBSA energy components and total binding energy estimates for all studied systems are summarized in Table 2. Because the NP-Pep and NR-Pep systems present multiple binding sites, the binding energetics were analyzed at 2 levels: Individual binding sites were evaluated independently to capture site-specific behavior, and average values were calculated to assess the overall binding tendency of each system. The results show favorable direct interactions between Ara h2 and all investigated receptors, as reflected by the negative gas-phase energies ($\Delta G_{\mathrm{gas}}$). In contrast, the solution-phase energy ($\Delta G_{\mathrm{sol}}$) is positive, indicating that solvation introduces an opposing desolvation penalty ($\Delta G_{\mathrm{sol}}>0$). Balancing the gas-phase and solvation contributions provides a comparative measure of interaction tendencies across systems, as reflected in the total binding energy estimates. These results highlight the important role of the solvent in modulating the interaction between Ara h2 and the receptors. The total energy estimates ($\Delta G_{\mathrm{tot}}$) indicate that peptide-functionalized NPs, particularly NR-Pep, exhibit more favorable MM-GBSA binding energy estimates compared to NP-Pep and IgE in several binding configurations, suggesting stronger relative interactions with Ara h2. However, due to the inherent limitations of the MM-GBSA method, these observations should be interpreted as comparative trends rather than absolute thermodynamic conclusions.

**Table 2.** MM-GBSA binding energy estimates (kcal/mol) for Ara h2 complexes with IgE, NP-Pep, and NR-Pep under NaCl and PBS conditions

System/solution environment	Binding site	ΔGgas	SD	ΔGsol	SD	ΔGtot	SD
Ara h2@IgE/NaCl	Single	−388.14	31.82	376.10	28.13	−12.04	6.46
Ara h2@IgE/PBS	Single	−436.25	27.05	402.16	24.77	−34.08	7.14
Ara h2@NP-Pep/NaCl	Site 1	−499.10	29.87	468.64	27.89	−30.46	6.48
	Site 2	−1,002.90	92.87	890.32	80.35	−112.58	17.54
	Site 3	−840.32	48.30	677.36	41.47	−162.96	14.03
	Site 4	−897.51	61.96	771.56	55.38	−125.95	12.75
	Site 5	−1,202.63	52.83	1,095.66	44.80	−106.98	13.42
Ara h2@NP-Pep/NaCl (Average)	–	−888.49	–	780.71	–	−107.79	–
Ara h2@NP-Pep/PBS	Site 1	−552.49	47.02	516.31	40.78	−36.18	10.64
	Site 2	−596.83	36.41	542.69	31.81	−54.14	8.63
	Site 3	−598.14	37.82	537.91	33.66	−60.23	8.47
	Site 4	−548.05	23.07	501.68	21.33	−46.37	6.09
	Site 5	−922.95	39.15	860.84	36.07	−62.11	7.90
Ara h2@NP-Pep/PBS (Average)	–	−643.69	–	591.93	–	−51.81	–
Ara h2@NR-Pep/NaCl	Site 1	−1,049.13	59.73	959.16	57.25	−89.97	9.83
	Site 2	−1,255.38	70.05	1,158.03	63.66	−97.35	11.38
	Site 3	−889.46	34.06	756.11	31.11	−133.35	10.07
	Site 4	−1,102.90	58.27	955.31	51.07	−147.58	14.55
	Site 5	−825.89	56.71	648.63	49.64	−177.26	12.78
	Site 6	−1,100.19	47.11	889.48	41.14	−210.72	13.56
	Site 7	−1,005.77	55.41	837.82	48.97	−167.96	12.40
	Site 8	−1,071.92	80.83	857.11	66.31	−214.81	20.64
Ara h2@NR-Pep/NaCl (average)	–	−1,037.33	–	882.71	–	−154.88	–
Ara h2@NR-Pep/PBS	Site 1	−1,274.55	51.60	1,197.94	45.88	−76.60	11.02
	Site 2	−877.05	83.03	843.08	77.18	−33.96	12.12
	Site 3	−796.59	50.04	704.04	46.62	−92.55	9.30
	Site 4	−927.79	74.74	813.78	66.45	−114.01	12.43
	Site 5	−847.93	66.73	687.48	57.93	−160.44	15.71
	Site 6	−1,110.15	66.91	966.97	60.22	−143.18	16.84
	Site 7	−911.57	80.14	764.63	72.45	−146.94	13.98
	Site 8	−1,153.45	54.08	928.14	47.06	−225.31	16.15
Ara h2@NR-Pep/PBS (average)	–	−987.89	–	863.76	–	−124.13	–

The total binding energy estimate ($\Delta G_{\mathrm{tot}}$) of the Ara h2@IgE system is −12.04±6.46 kcal/mol in NaCl solution, decreasing to −34.08±7.14 kcal/mol in buffer, making it more favorable. Considering the strong gas-phase energy in buffer ($\Delta G_{\mathrm{gas}}$ = −436.25±27.05 kcal/mol) for this complex, the role of the unfavorable solvation contribution is highlighted by the dominant role of ElE and vdW interactions in stabilizing the Ara h2@IgE complex under buffered conditions. These data indicate that the native Ara h2@IgE interaction is energetically weak and solvent-sensitive, consistent with the limited hydrogen bonding, lower contact numbers, higher solvent exposure (SASA), and larger hydration shell populations observed earlier. The MM-GBSA binding energy estimates for these systems appear less favorable than expected for typical antibody–antigen interactions, which may arise from modeling limitations. However, the Fab fragment of IgE captures the key antigen-binding interface; the model does not include the full IgE antibody or glycosylation effects, which may influence binding stability. Furthermore, the complex was generated using protein–protein docking and may not fully reproduce the native binding configuration.

These energies are getting substantially stronger when Ara h2 is bound to the NP-Pep system. $\Delta G_{\mathrm{tot}}$ values for Ara h2@NP-Pep range from approximately −30 to −163 kcal/mol in physiological conditions, reflecting large variability in interfacial complementarity. The most negative free energy is observed with −1,002.90 kcal/mol in the gas phase. Under buffer conditions, NP-Pep binding becomes more uniform across sites, with $\Delta G_{\mathrm{tot}}$ values clustering between −36 and −54 kcal/mol. Overall, comparing average values confirms that Ara h2 binding to NP-Pep sites in NaCl solution (−108.99) is stronger than in buffer (−49.23), indicating that buffer conditions substantially reduce the ElE driving force for NP-Pep binding. These results are in agreement with earlier dynamical and interactional analyses.

The binding energies reported in this study are evaluated in a pairwise manner and therefore do not explicitly capture cooperative effects such as cross-linking or clustering. The NP-Pep and NR-Pep systems exhibit heterogeneous binding behavior, as evidenced by multiple binding orientations and interaction sites. Accordingly, the reported average binding energies are presented as summary descriptors of overall trends rather than precise thermodynamic quantities. The enhanced binding observed for the NP systems may partially arise from multivalent effects associated with NP geometry and surface functionalization. Therefore, the calculated binding energies should be interpreted as relative indicators of interaction strength rather than absolute measures of binding affinity. The most favorable relative binding energy estimates are obtained when Ara h2 binds to the NR-Pep system. In NaCl solution, the average total binding energy ($\Delta G_{\mathrm{tot}}$) reaches −154.88 kcal/mol. Examination of the site-specific binding energies for this system reveals substantial variability among the 8 binding sites, with $\Delta G_{\mathrm{tot}}$ values ranging from approximately −90 to −215 kcal/mol in NaCl solution. Several sites exhibit exceptionally strong binding in NaCl solution, including values of −210.72 and −214.81 kcal/mol. Strong binding is also preserved under buffer conditions with the most favorable binding site with $\Delta G_{\mathrm{tot}}$ value of −225.31 kcal/mol. The average binding free energy across all NR-Pep sites in buffer is −124.13 kcal/mol. Therefore, NR-Pep forms robust, energetically stable complexes with Ara h2 across different solvent conditions, despite site-specific variability. It is worth mentioning that NR-Pep in NaCl solution exhibits the most favorable MM-GBSA binding energy estimates to Ara h2, whereas NP-Pep in buffer shows the weakest binding. Despite NP-Pep achieving strong interfacial engagement, NR-Pep exhibits stable, favorable binding through a balanced combination of ElE, vdW, and H-bond interactions. The solvent-sensitive ElE interactions and extensive surface masking of NP-Pep lead to a high contact density and increased heterogeneity, whereas NR-Pep maintains consistent energetics and moderate solvent accessibility across conditions.

In total, the MM-GBSA energy values align with the full set of structural, energetic, and interfacial parameters. We can conclude that more favorable MM-GBSA binding energy estimates correlate with reduced SASA, diminished hydration shells, increased contact numbers, and enhanced ElE stabilization. The higher binding favorability of the designed peptide-functionalized NPs compared with IgE confirms their effectiveness in competing with the human receptor for Ara h2 binding. Among all proposed receptors, NR-Pep demonstrates the most robust performance in both NaCl solution and PBS buffer environments, consistently standing out in combined structural stability and energetic evaluations. The obtained results support the enhanced interaction capability of peptide-functionalized NPs, particularly NR-Pep, relative to the native IgE system. These results demonstrate the importance of considering not only binding strength but also energetic balance and solvent robustness in the design of NP-based binding systems.

## Conclusion

MD simulations were used to investigate the affinities of IgE, NP-Pep, and NR-Pep for the Ara h2 peanut allergen. Based on the results, designed peptide-functionalized NP systems influence the structural stability of Ara h2. Free energy analysis reveals that Ara h2 forms significant interactions with both NP-Pep and NR-Pep. Moreover, these nanomaterials can modulate the protein’s dynamic behavior through interactions with Ara h2. ElE and vdW interactions between Ara h2 and the binding sites of receptors play a key role in stabilizing the corresponding complexes. The strength of Ara h2 interactions with NP-Pep and NR-Pep, as well as their impact on protein dynamics, differs due to the variety of amino acid residues involved at the interaction sites. Ara h2 forms a more stable complex with NR-Pep because of stronger ElE and vdW interactions compared to IgE and NP-Pep. Furthermore, based on calculated binding energies, NR-Pep and NP-Pep exhibit higher sensitivity and affinity toward Ara h2 than IgE. Overall, these analyses suggest that NR-Pep has significant potential as an agent for designing new sensors to detect Ara h2. The solvent effect study confirmed that different solvent conditions may significantly alter the conformational behavior and binding site properties. These observations confirm the critical role of AuNPs shape and ionic environment in modulating Ara h2 dynamics and interactions. This study provides fundamental insights for developing peptide-based binding systems that effectively mimic IgE recognition. Through this research, NP-Pep and NR-Pep systems are introduced as promising candidates for targeting Ara h2 due to their higher binding affinity and potential to interact with the allergen.

## Data Availability

Molecular dynamics simulation data generated in this study have been deposited in the Zenodo repository under the identifier 10.5281/zenodo.20055781 and will be publicly available upon publication of the article. Additional data not included in the public repository are available from the corresponding author upon reasonable request. No restrictions apply to data availability.
