# Supplementary material for: Gold Nanoparticle-Based Systems for Targeting the Ara h2 Allergen: A Molecular Modeling Approach
Source: Comput Struct Biotechnol J. 2026 May 11;35(3):0088. doi: 10.34133/csbj.0088 (PMC13632937; doi:10.34133/csbj.0088)
Supplement: Supplementary file 1 [file csbj.0088.f1.pdf]

Supplementary Information

Gold Nanoparticle–Based Systems for Targeting the Ara h2 Allergen: A Molecular Modeling Approach

Aliyeh Mehranfar[1], Akhilesh Kumar Shakya[1,2], Roland Faller[1]

[1]Department of Chemical Engineering, Texas Tech University, Lubbock, TX 79409, USA
[2]Bioengineering, Whitacre College of Engineering, Texas Tech University, Lubbock, TX 79409, USA

amehranf@ttu.edu, akhilesh.shakya@ttu.edu, Roland.Faller@ttu.edu

February 2026

FIG. S1: Structural stability and flexibility analysis of the peptide during the 500 ns MD simulation. (A) RMSD, (B) RMSF, and (C) polar solvent-accessible surface area (SASA) profiles.

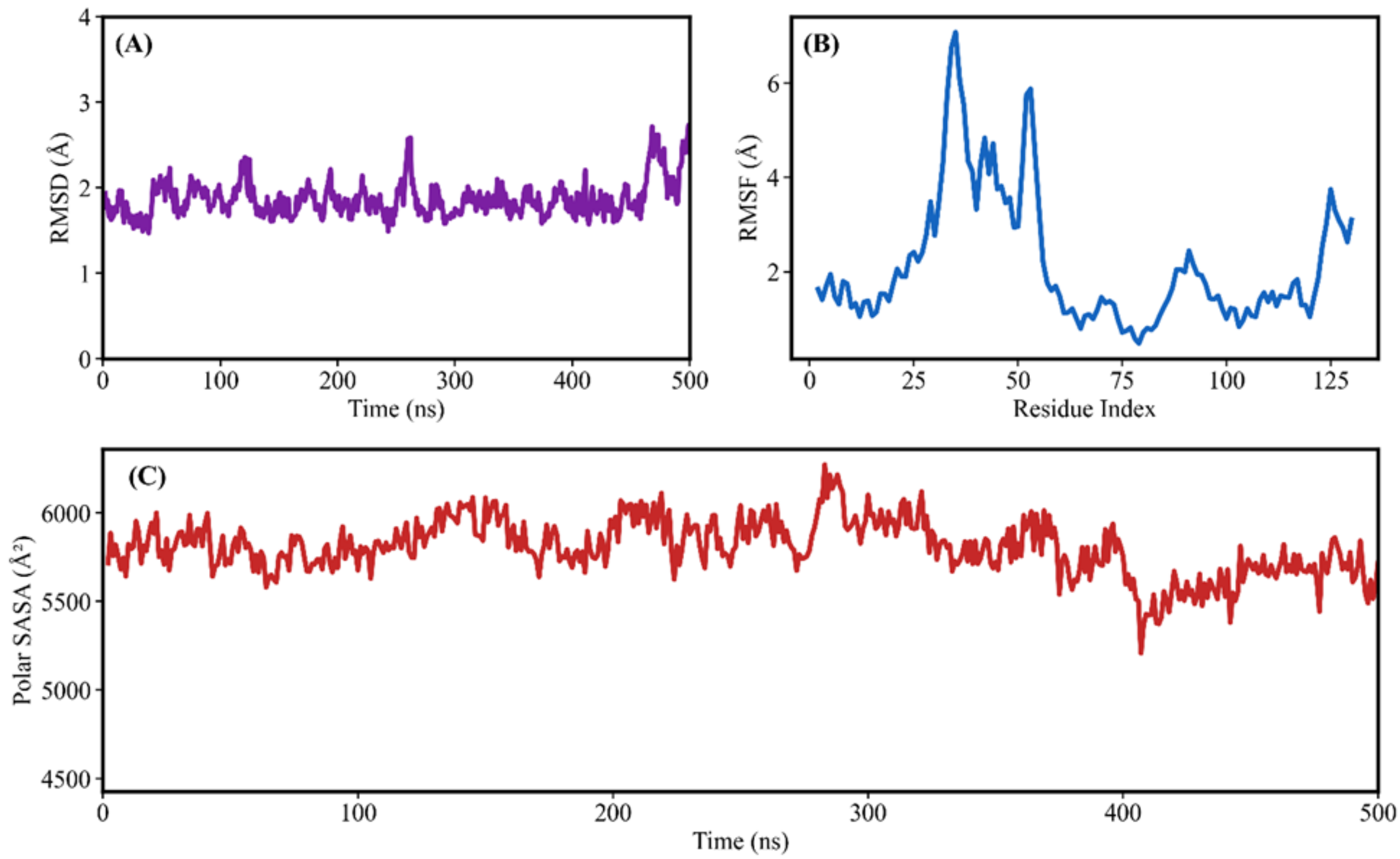

FIG. S2: Time resolved secondary structure evolution of the Aar h2 residues over the 500 ns MD simulation. The color scale denotes different secondary structure elements, including -helix, 3–10 helix, -helix, turn, bend, -bridge, extended structure, and disordered regions (none).

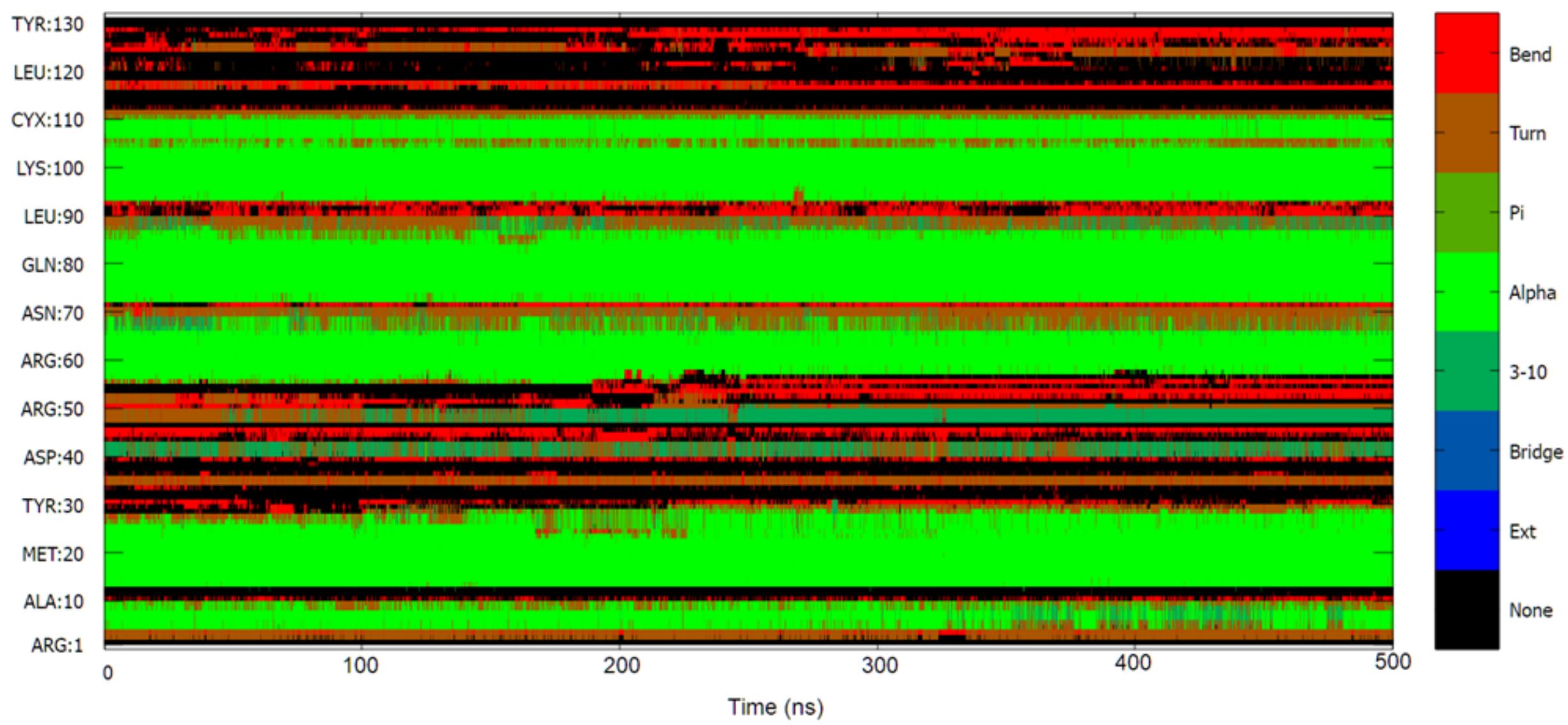

FIG. S3: Time resolved secondary structure evolution of the IgE receptor during 500 ns MD simulations under two different conditions: (A) NaCl solution and (B) PBS buffer. Color coding indicates different secondary structure elements, including α-helix, 3–10 helix, π-helix, turn, bend, β-bridge, extended structure, and disordered (none), as indicated in the legend.

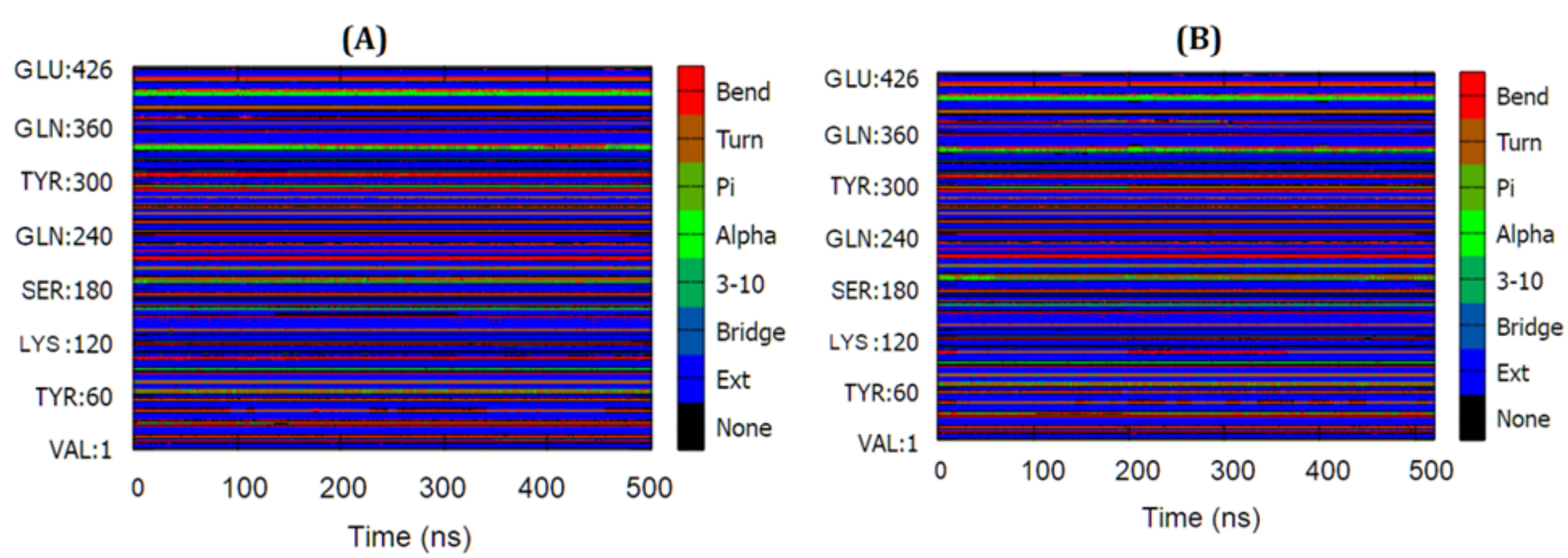

TABLE S1: Total solvent accessible surface area (Total SASA) of Ara h2 within different binding receptors

System	Solution environment	Average± Std. Dev. (Å²)	Peak (Å²)
Ara h2@IgE	NaCl	8058±331	7852
Ara h2@IgE	PBS	7734±531	7343
Ara h2@NP-Pep	NaCl	7642±385	7633
Ara h2@NP-Pep	PBS	6791±787	6276
Ara h2@NR-Pep	NaCl	7732±385	7706
Ara h2@NR-Pep	PBS	7555±457	7431

TABLE S2: Polar solvent-accessible surface area (Polar SASA) of Ara h2 within different binding receptors

System	Solution environment	Average± Std. Dev. (Å²)	Peak (Å²)
Ara h2@IgE	NaCl	5076±255	4943
Ara h2@IgE	PBS	4929±371	4665
Ara h2@NP-Pep	NaCl	4829±259	4749
Ara h2@NP-Pep	PBS	3743±610	3250
Ara h2@NR-Pep	NaCl	4883±259	4853
Ara h2–NR-Pep	PBS	4765±288	4737

TABLE S3: Hydration, hydrogen bonding, and interaction energies of Ara h2 in the complex with IgE under NaCl solution and buffer conditions

Property	Solution environment	Average ± SD
Water shell (1st shell)	NaCl	636.25 ± 21.06
Water shell (1st shell)	PBS	615.57 ± 17.33
H-bonds	NaCl	1.68 ± 1.26
H-bonds	PBS	3.40 ± 2.41
Electrostatic energy (kcal/mol)	NaCl	-241.52 ± 56.96
Electrostatic energy (kcal/mol)	PBS	-284.11 ± 57.66
van der Waals energy (kcal/mol)	NaCl	-43.50 ± 9.32
van der Waals energy (kcal/mol)	PBS	-77.41 ± 8.04

FIG. S4: Comparison of Ara h2 binding orientations across different systems after 500 ns of MD simulations in NaCl solution. (A) Ara h2@IgE reference complex. (B–F) Representative binding configurations of Ara h2 at NP-Pep binding sites. All structures were aligned using the Cα atoms of Ara h2 in the IgE-bound complex as the reference to enable direct comparison of binding orientations, with the reference structure shown in transparent grey.

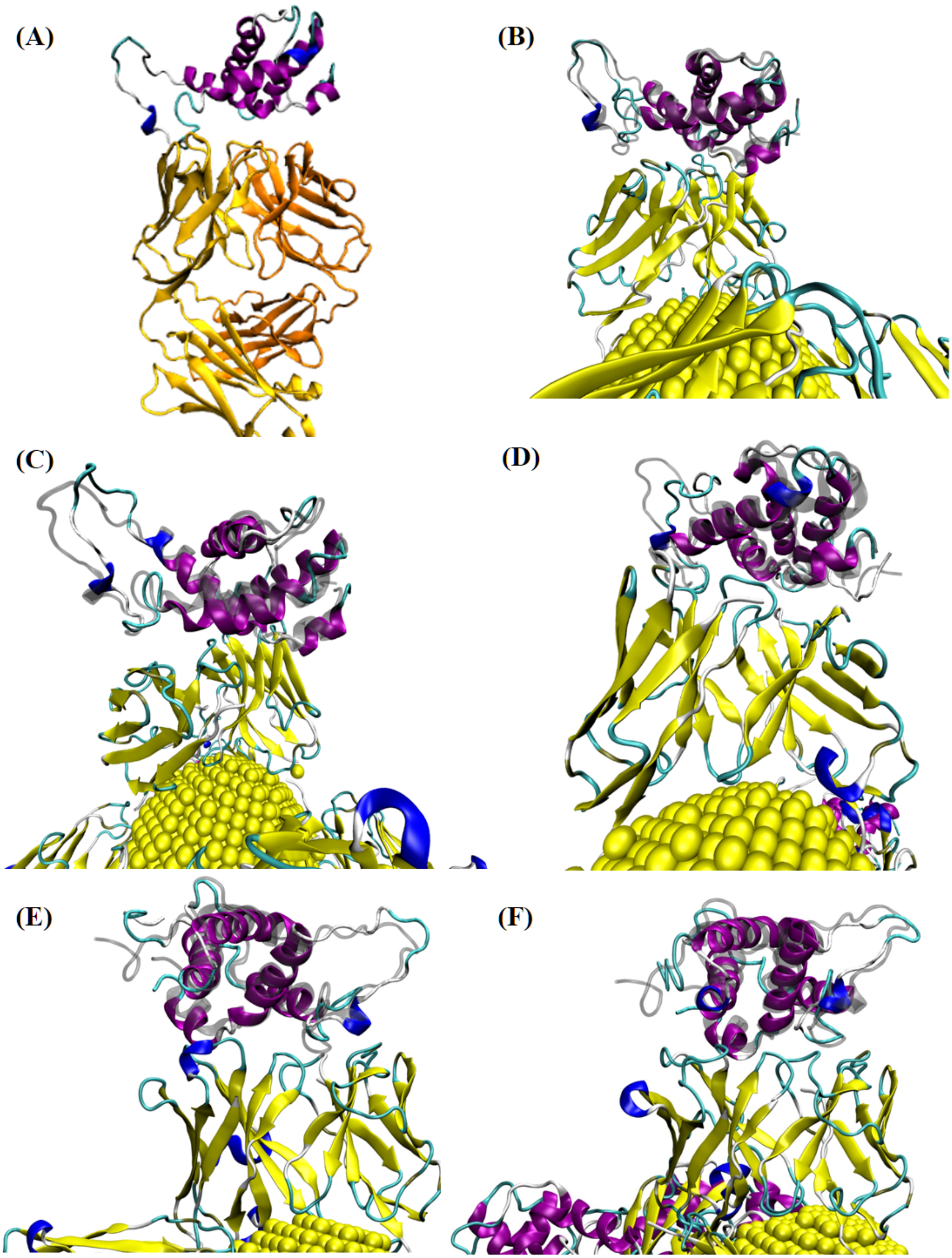

FIG. S5: (A-H) Representative binding configurations of Ara h2 in complex with NR-Pep binding sites after 500 ns of MD simulations in NaCl solution. Structures were aligned using the Cα atoms of Ara h2 in the IgE-bound complex as the reference to enable direct comparison of binding orientations, with the reference structure shown in transparent grey.

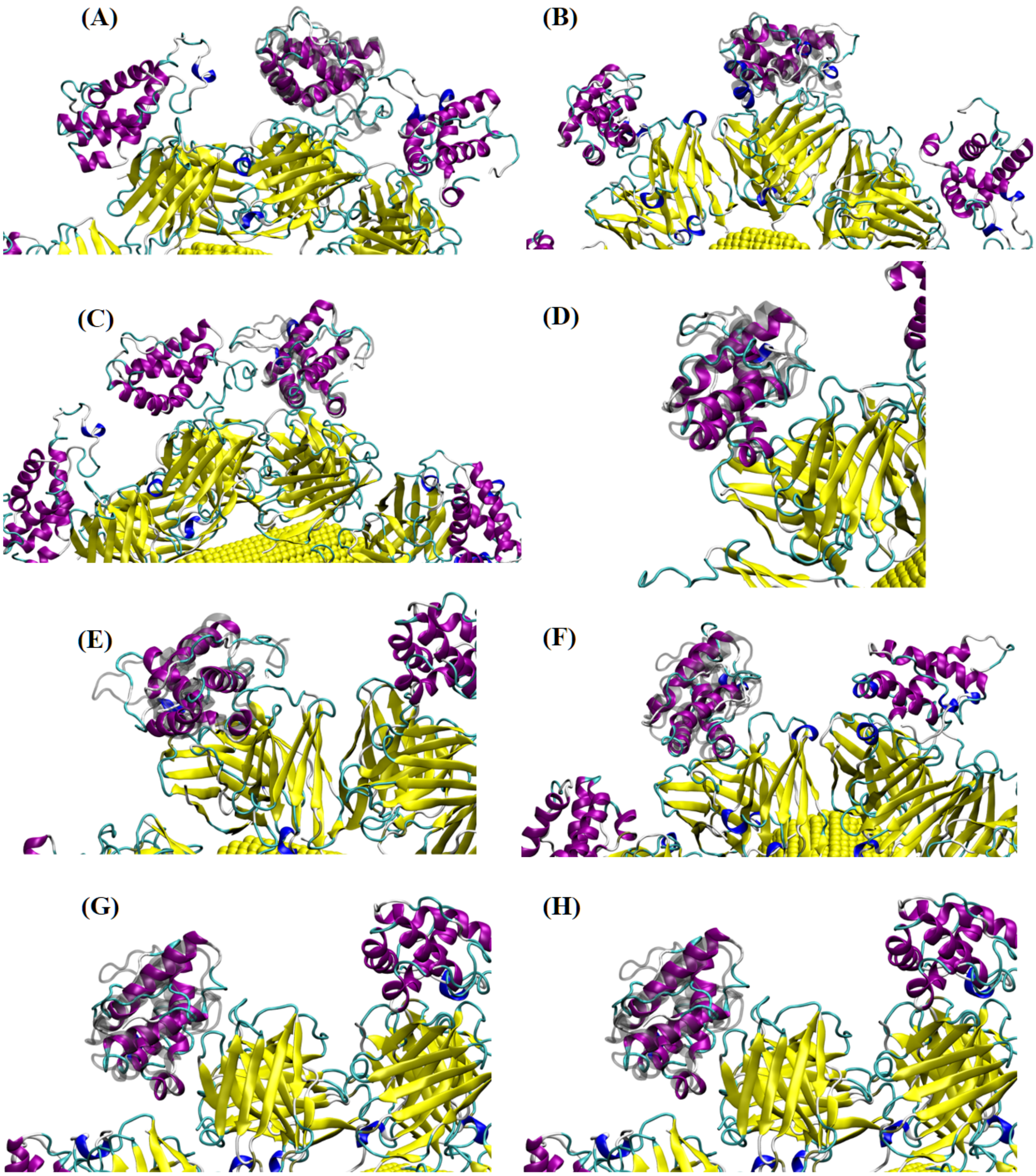

FIG. S6: Comparison of Ara h2 binding orientations across different systems after 500 ns of MD simulations in PBS buffer. (A) Ara h2@IgE reference complex. (B–F) Representative binding configurations of Ara h2 at NP-Pep binding sites. All structures were aligned using the Cα atoms of Ara h2 in the IgE-bound complex as the reference to enable direct comparison of binding orientations, with the reference structure shown in transparent grey.

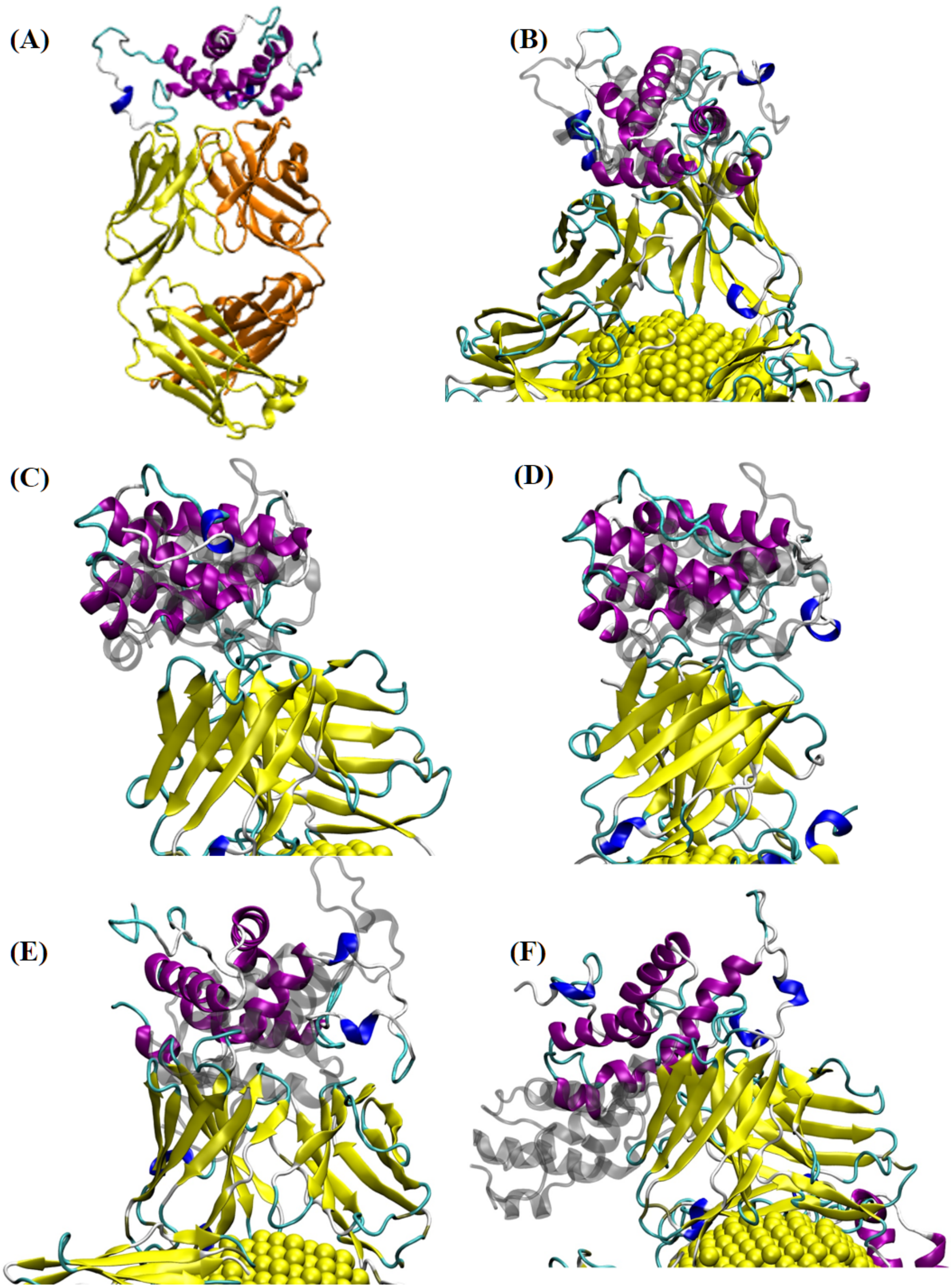

FIG. S7: (A-H) Representative binding configurations of Ara h2 in complex with NR-Pep binding sites after 500 ns of MD simulations in PBS buffer. Structures were aligned using the Cα atoms of Ara h2 in the IgE-bound complex as the reference to enable direct comparison of binding orientations, with the reference structure shown in transparent grey.

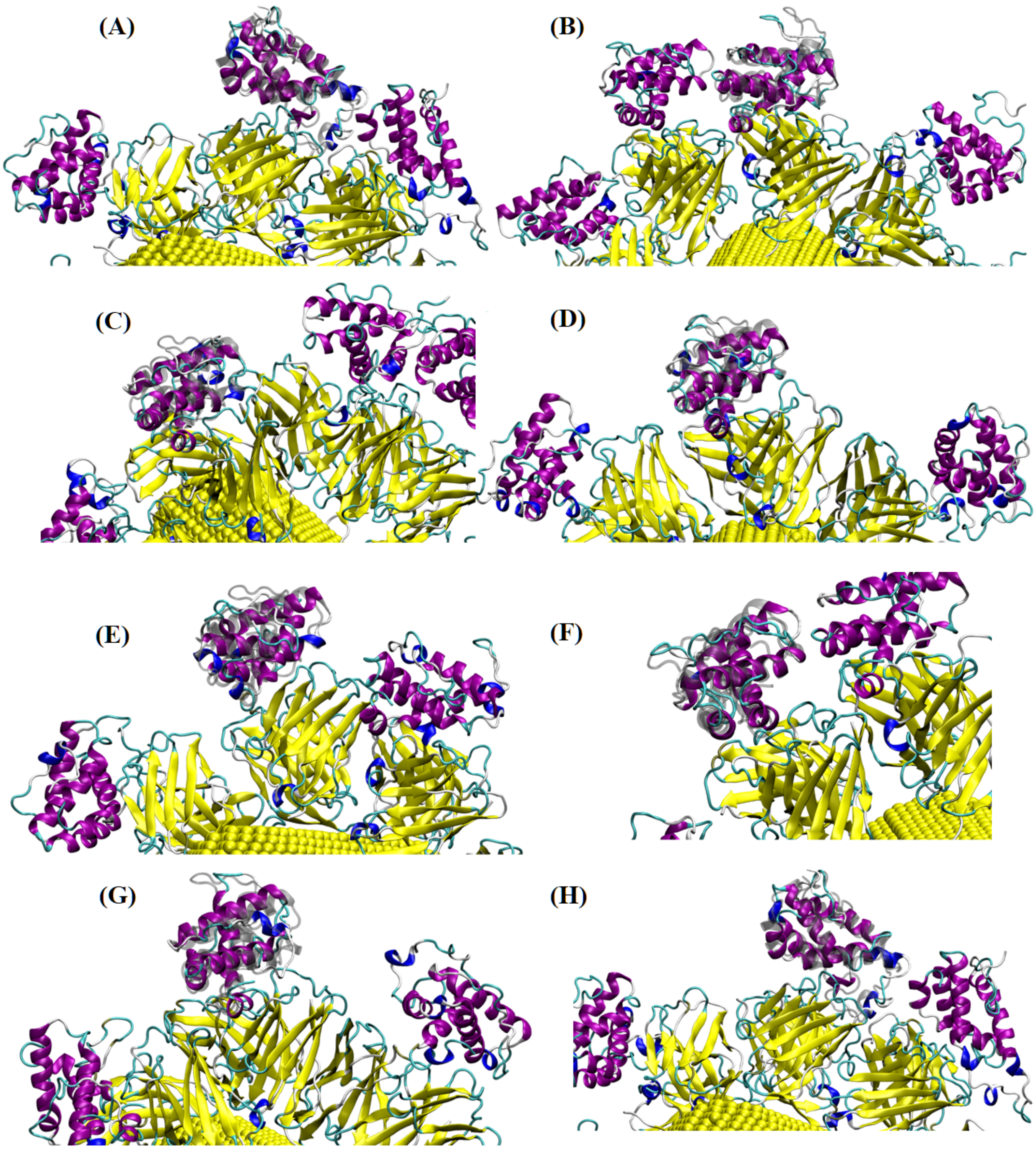

FIG. S8: (A) RMSD of Ara h2@NP- Pep in NaCl solution, (B) Rg of Ara h2@NP-Pep in NaCl solution, (C) RMSD of Ara h2@NP-Pep in PBS buffer (D) Rg of Ara h2@NP-Pep in PBS buffer (E) RMSD of Ara h2@NR- Pep in NaCl solution (F) Rg of Ara h2@NR-Pep in NaCl solution (G) RMSD of Ara h2@NR-Pep in PBS buffer (H) Rg of Ara h2@NR-Pep in PBS buffer. Solid lines represent the average, while the shaded regions indicate different Ara h2 fluctuations along the trajectories.

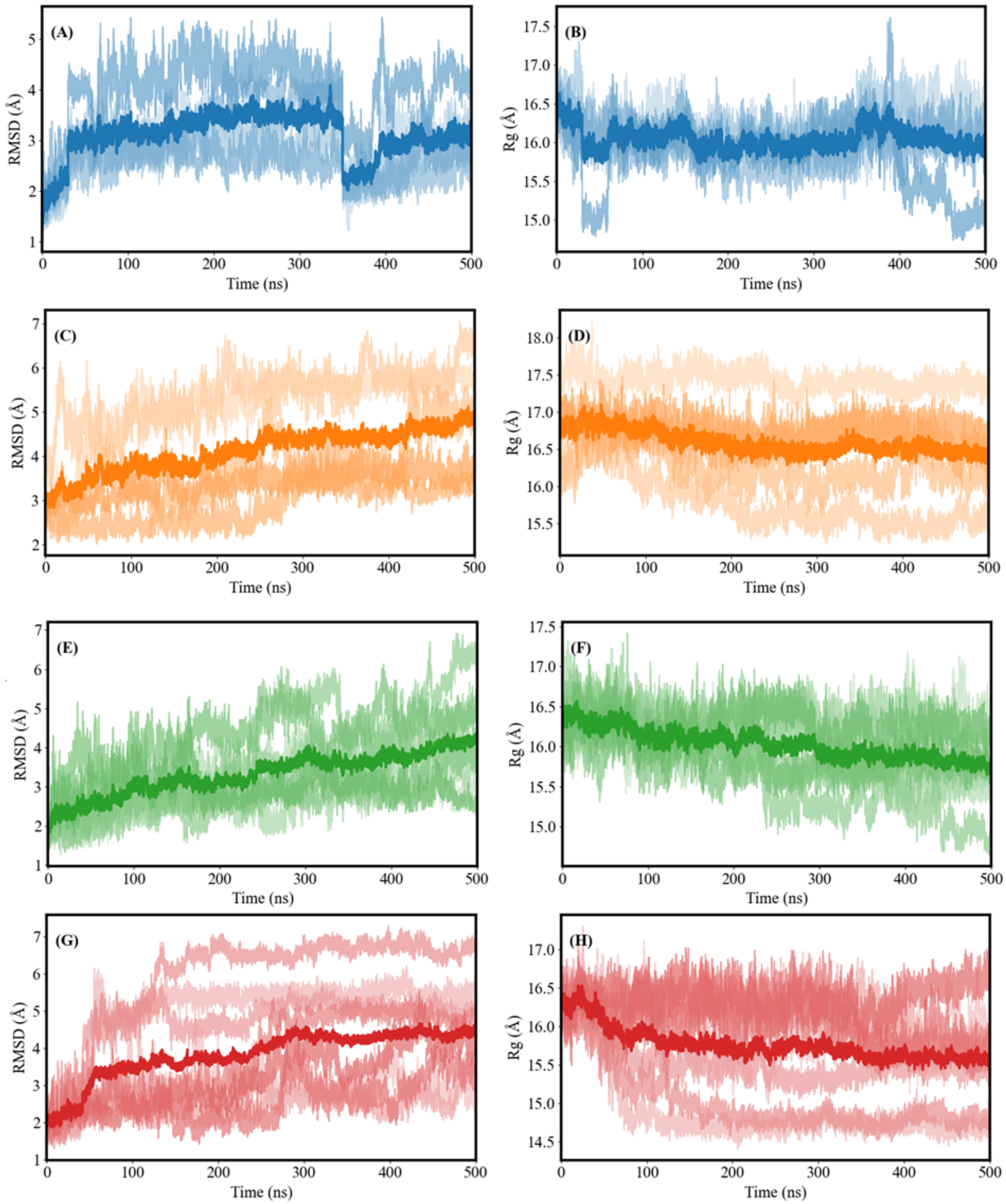

FIG. S9: Root-mean-square fluctuation (RMSF) profiles of the four Ara h2 structures: (A) residues 230–359, (B) residues 589–718, (C) residues 942–1071, and (D) residues 1299–1428. RMSF values were calculated from the MD trajectories for NP–Pep and NR–Pep complexes simulated in NaCl solution and PBS buffer.

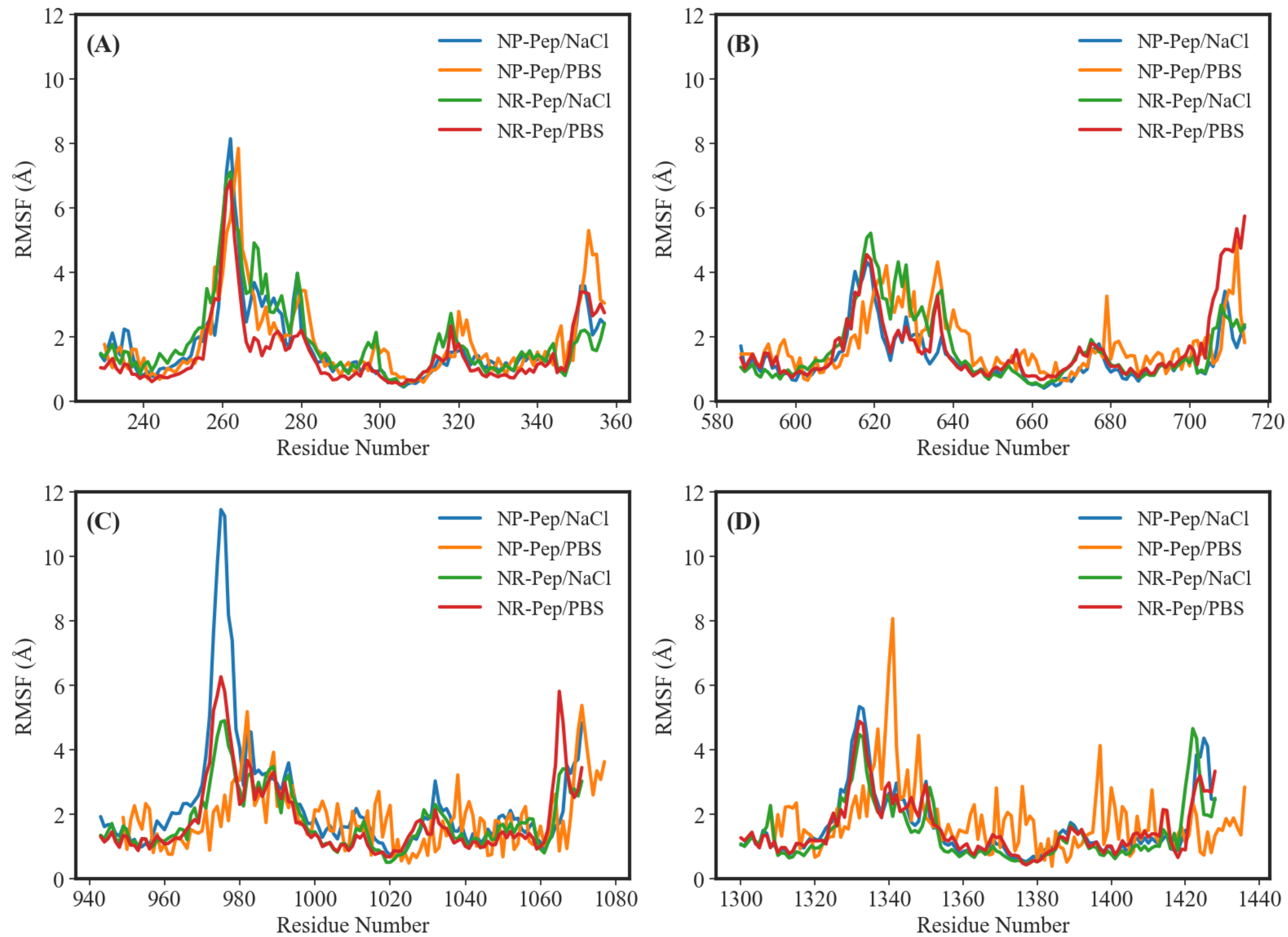

FIG. S10: Average number of contacts formed between the receptor and Ara h2 under in NaCl solution and PBS buffer environments, as indicated on the x-axis. Error bars correspond to the standard deviation.

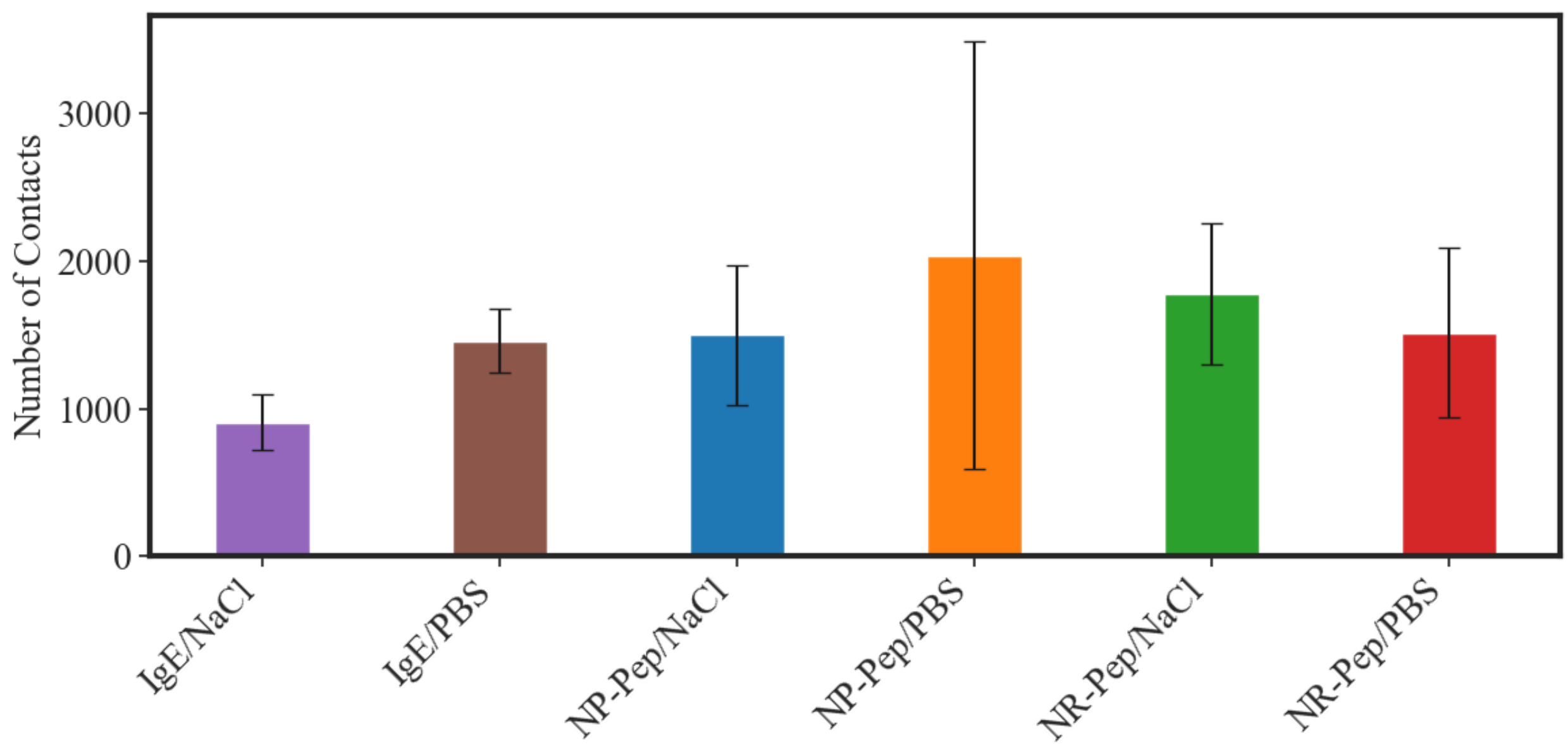

FIG. S11: Interaction properties of Ara h2 and IgE under NaCl solution and PBS buffer conditions. (A) The distributions of first-shell hydration water molecules, (B) frequency of hydrogen bonds, (C) electrostatic interaction energy, and (D) van der Waals interaction energy. Histograms represent sampled values from the trajectories, and solid curves denote kernel density estimates. Average peak values for each distribution are reported in Table S3.

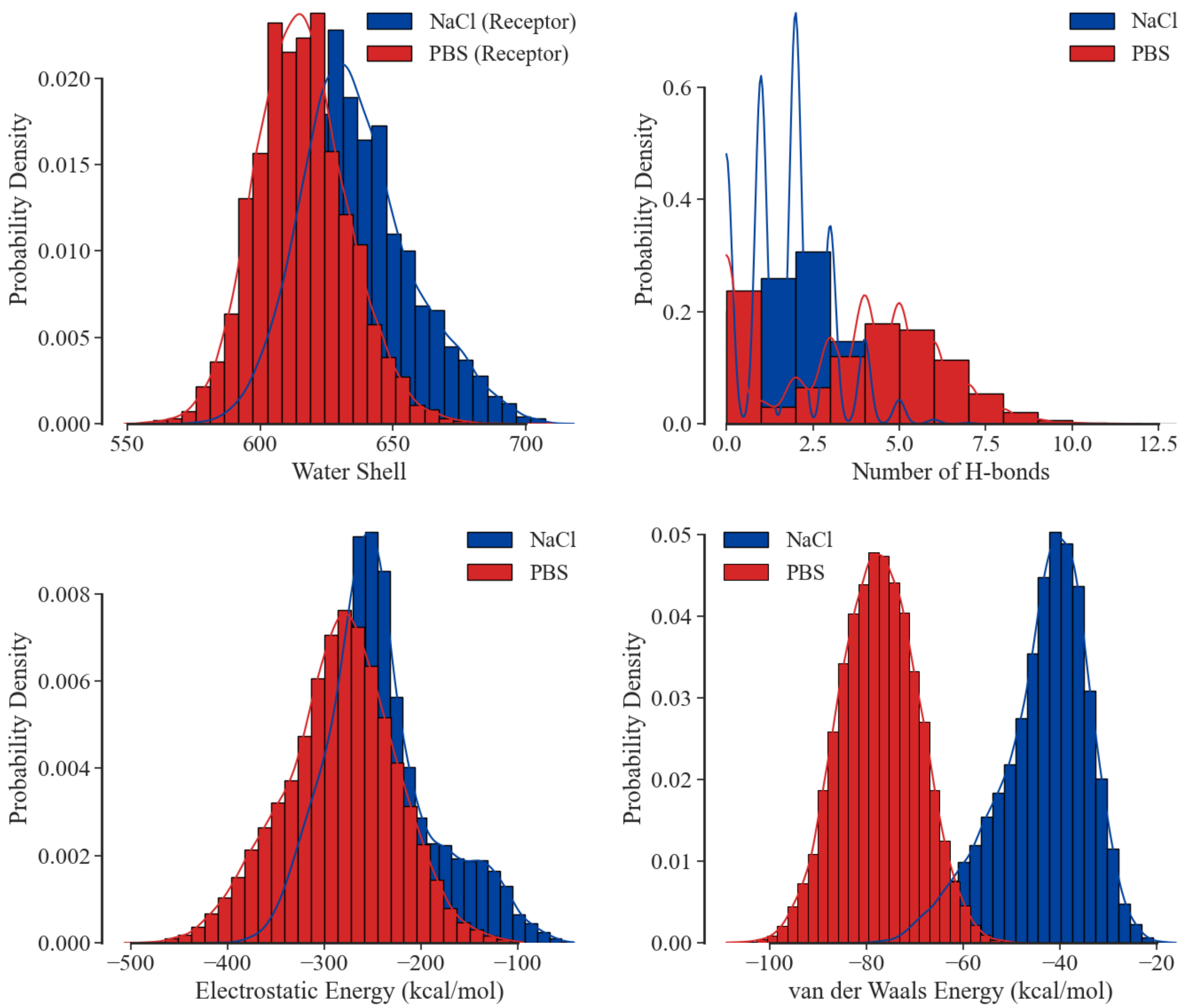

FIG. S12: Average electrostatic interaction energies (ElE) between Ara h2 and IgE, NP-Pep, and NR-Pep under NaCl solution and PBS buffer conditions, obtained from linear interaction energy (LIE) analysis. The ElE contributions are reported for individual Ara h2 replicas (Ara h2_1–Ara h2_8). Error bars represent standard deviations calculated over the simulation trajectories.

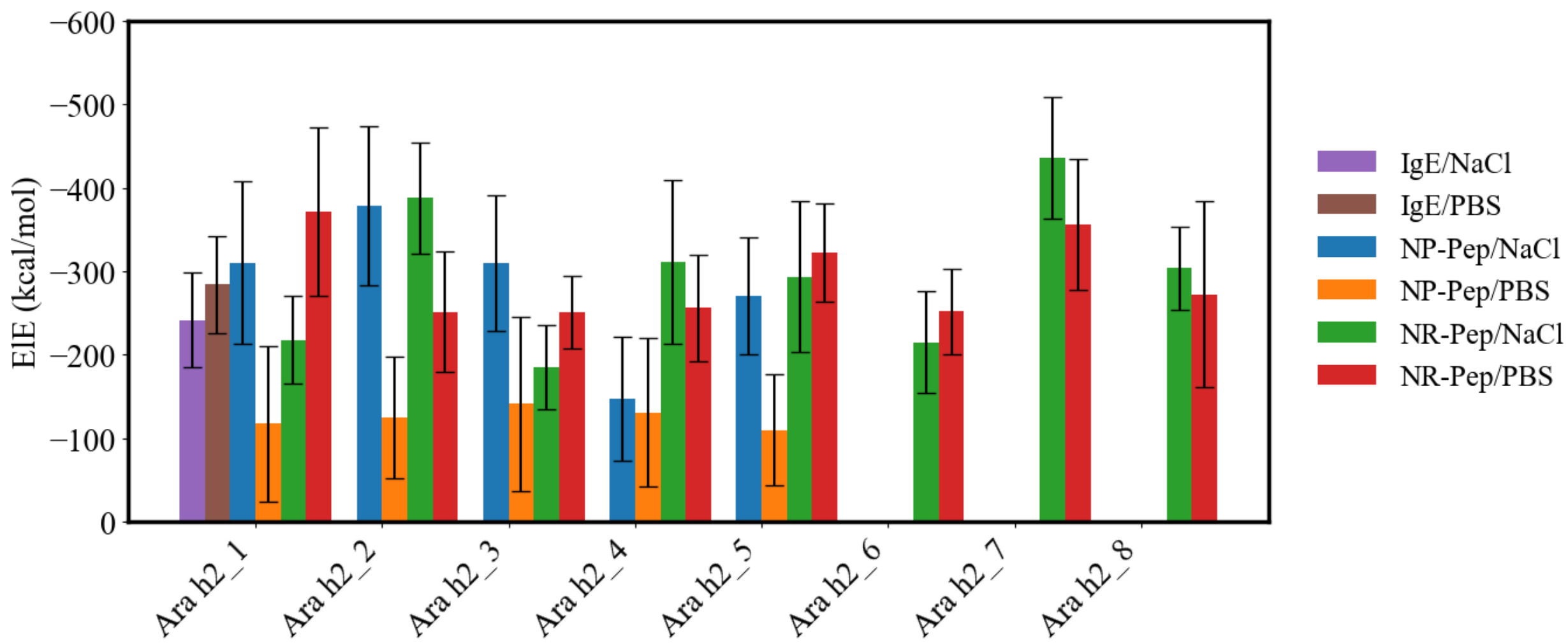

FIG. S13: Average van der Waals (vdW) interaction energies between Ara h2 and IgE, NP-Pep, and NR-Pep under NaCl solution and PBS buffer conditions, obtained from linear interaction energy (LIE) analysis. The vdW contributions are reported for individual Ara h2 replicas (Ara h2_1–Ara h2_8). Error bars represent standard deviations calculated over the simulation trajectories.

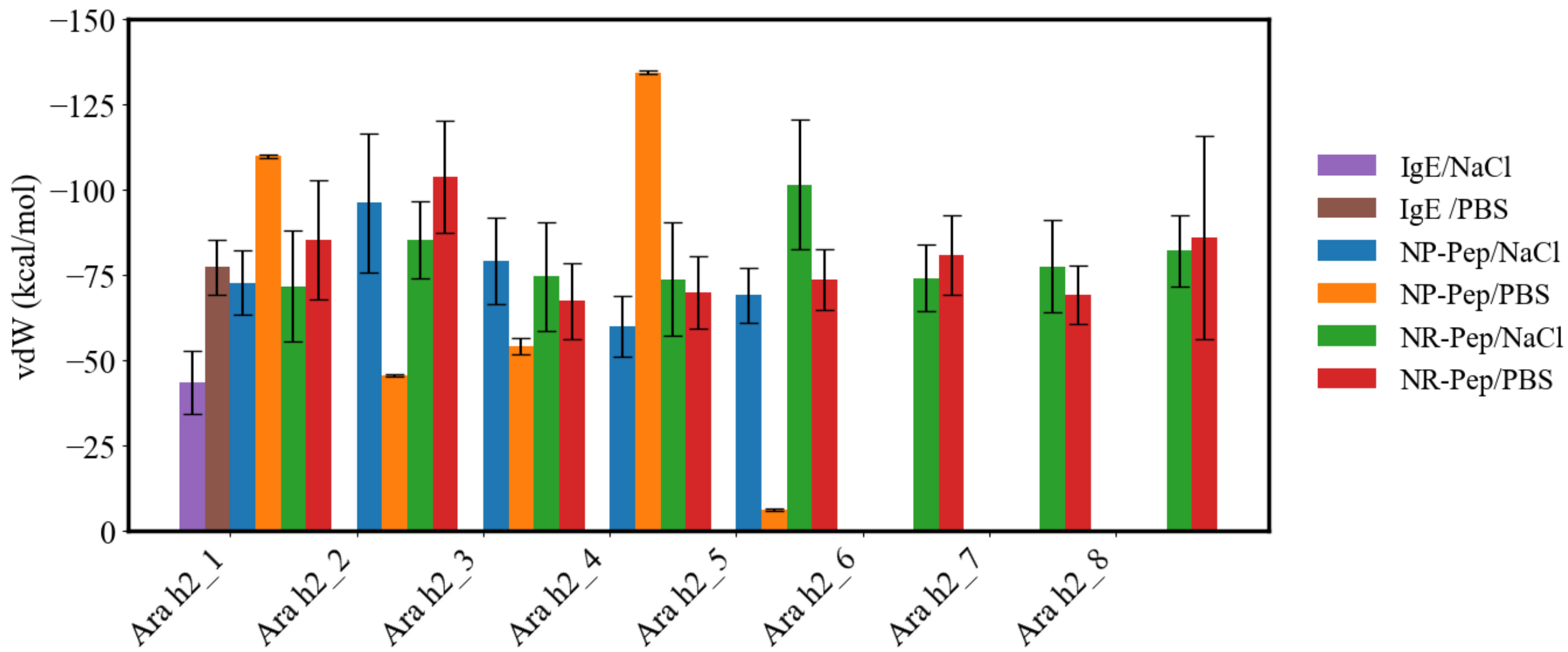

TABLE S4: Site-resolved electrostatic (ElE) and van der Waals (vdW) interaction energies for Ara h2 complexes

System	Solution environment	Ara h2 Site	ElE (kcal/mol)	vdW (kcal/mol)
Ara h2@IgE	NaCl	Ara h2_1	-241.52 ± 56.96	-43.50 ± 9.32
Ara h2@IgE	PBS	Ara h2_1	-284.11 ± 57.66	-77.41 ± 8.04
Ara h2@NP-Pep	NaCl	Ara h2_1	-310.32 ± 97.22	-72.69 ± 9.42
		Ara h2_2	-379.14 ± 95.44	-96.18 ± 20.34
		Ara h2_3	-310.15 ± 80.82	-79.23 ± 12.69
		Ara h2_4	-147.02 ± 74.15	-59.99 ± 9.00
		Ara h2_5	-270.25 ± 69.85	-69.09 ± 8.06
Ara h2@NP-Pep	PBS	Ara h2_1	-117.33 ± 92.96	-109.78 ± 0.50
		Ara h2_2	-124.54 ± 72.68	-45.59 ± 0.50
		Ara h2_3	-141.09 ± 104.64	-54.26 ± 2.45
		Ara h2_4	-131.26 ± 88.34	-134.34 ± 0.44
		Ara h2_5	-110.05 ± 67.04	-6.15 ± 0.38
Ara h2@NR-Pep	NaCl	Ara h2_1	-217.56 ± 52.47	-71.69 ± 16.26
		Ara h2_2	-388.01 ± 66.92	-85.41 ± 11.29
		Ara h2_3	-185.39 ± 49.93	-74.59 ± 15.86
		Ara h2_4	-311.62 ± 98.41	-73.79 ± 16.50
		Ara h2_5	-293.27 ± 90.42	-101.49 ± 19.05
		Ara h2_6	-215.31 ± 61.00	-74.08 ± 9.80
		Ara h2_7	-436.16 ± 72.94	-77.55 ± 13.49
		Ara h2_8	-303.74 ± 50.04	-82.12 ± 10.40
Ara h2@NR-Pep	PBS	Ara h2_1	-371.85 ± 100.60	-85.34 ± 17.47
		Ara h2_2	-251.69 ± 72.23	-103.69 ± 16.48
		Ara h2_3	-251.40 ± 43.23	-67.39 ± 11.19
		Ara h2_4	-255.99 ± 63.36	-69.99 ± 10.65
		Ara h2_5	-322.49 ± 58.35	-73.61 ± 8.81
		Ara h2_6	-252.24 ± 51.13	-80.85 ± 11.76
		Ara h2_7	-356.05 ± 78.43	-69.20 ± 8.51
		Ara h2_8	-272.69 ± 111.89	-86.05 ± 29.76
